# Differential prioritization between relevance and redundancy in correlation-based feature selection techniques for multiclass gene expression data

**DOI:** 10.1186/1471-2105-7-320

**Published:** 2006-06-23

**Authors:** Chia Huey Ooi, Madhu Chetty, Shyh Wei Teng

**Affiliations:** 1 Gippsland School of Information Technology, Monash University, Churchill, VIC 3842, Australia

## Abstract

**Background:**

Due to the large number of genes in a typical microarray dataset, feature selection looks set to play an important role in reducing noise and computational cost in gene expression-based tissue classification while improving accuracy at the same time. Surprisingly, this does not appear to be the case for all multiclass microarray datasets. The reason is that many feature selection techniques applied on microarray datasets are either rank-based and hence do not take into account correlations between genes, or are wrapper-based, which require high computational cost, and often yield difficult-to-reproduce results. In studies where correlations between genes are considered, attempts to establish the merit of the proposed techniques are hampered by evaluation procedures which are less than meticulous, resulting in overly optimistic estimates of accuracy.

**Results:**

We present two realistically evaluated correlation-based feature selection techniques which incorporate, in addition to the two existing criteria involved in forming a predictor set (relevance and redundancy), a third criterion called the degree of differential prioritization (DDP). DDP functions as a parameter to strike the balance between relevance and redundancy, providing our techniques with the novel ability to differentially prioritize the optimization of relevance against redundancy (and vice versa). This ability proves useful in producing optimal classification accuracy while using reasonably small predictor set sizes for nine well-known multiclass microarray datasets.

**Conclusion:**

For multiclass microarray datasets, especially the GCM and NCI60 datasets, DDP enables our filter-based techniques to produce accuracies better than those reported in previous studies which employed similarly realistic evaluation procedures.

## Background

The aim of feature selection is to form, from all available features in a dataset, a relatively small subset of features capable of producing the optimal classification accuracy. This subset is called the *predictor set*. The following are past and current stances on the use of feature selection for multiclass tissue classification:

• Feature selection does not aid in improving classification accuracy [1,2], at least not as much as the type of classifier used.

• Feature selection is often rank-based, and is implemented mainly with the intention of merely reducing cost/complexity of subsequent computations (since the transformed dataset is smaller), rather than also finding the feature subset which best explains the dataset [1,3].

• Studies proposing feature selection techniques with sophistication above that of rank-based techniques resort to an evaluation procedure which is prone to giving overly optimistic estimate of accuracy, but has the advantage of costing less computationally than procedures which yield a more realistic estimate of accuracy [4-7].

In short, there are three ways in which feature selection has been, and still is regarded for multiclass microarray datasets: 1) should not be considered at all, 2) as simple rank-based methods for dataset truncation, and finally, 3) as more complicated methods with sound theoretical foundation, but with doubtful empirical results.

A feature selection technique is made of two components: the predictor set scoring method (which evaluates the goodness of a candidate predictor set); and the search method (which searches the gene subset space for the predictor set based on the scoring method). The technique becomes wrapper-based when classifiers are invoked in the predictor set scoring method. Otherwise, the technique is filter-based. Filter-based techniques, which are the focus of this study, have several advantages over wrapper-based techniques: 1) Filter-based techniques cost less computationally than wrapper-based techniques. 2) Filter-based techniques are not classifier-specific. 3) More importantly, unlike the typical 'black-box' trait of wrapper-based techniques, filter-based techniques provide a clear picture of why a certain feature subset is chosen as the predictor set through the use of scoring methods in which inherent characteristic(s) of the predictor set (and not its prediction ability) is optimized. The last advantage is particularly crucial since the predictor set scoring method in a filter-based technique can explain the prediction ability of the predictor set, whereas in a wrapper-based technique, the score of goodness of the predictor set is its prediction ability itself, and hence the term 'black-box'.

An important principle behind most filter-based feature selection techniques can be summarized by the following statement: *A good predictor set should contain features highly correlated with the target class distinction, and yet uncorrelated with each other *[8]. The predictor set attribute referred to in the first part of this statement, 'relevance', is the backbone of simple rank-based feature selection techniques. The aspect alluded to in the second part, 'redundancy', refers to pairwise relationships between all pairs of genes in the predictor set.

Previous studies [4,8] have based their feature selection techniques on the concept of relevance and redundancy having *equal *importance in the formation of a good predictor set. We call the predictor set scoring methods used in such correlation-based feature selection techniques *equal-priorities scoring methods*. On the other hand, Guyon and Elisseeff (2003) [9] demonstrated using a 2-class problem that seemingly redundant features may improve the discriminant power of the predictor set instead, although it remains to be seen how this scales up to multiclass domains with thousands of features. A study was implemented on the effect of varying the importance of redundancy in predictor set evaluation in [10]. However, due to its use of a relevance score that is inapplicable to multiclass problems, the study was limited to only binary classification.

From here, we can state the three levels of filter-based feature selection for multiclass tumor classification as follows: 1) no selection, 2) select based on relevance alone, and finally, 3) select based on relevance **and **redundancy. Thus, currently, relevance and redundancy are the two existing criteria which have ever been used in predictor set scoring methods for multiclass tumor classification.

### Contributions of this study

We propose to go one step further, by introducing a third criterion: the relative importance placed between relevance and redundancy. We call this criterion the **d**egree of **d**ifferential **p**rioritization (DDP). DDP compels the search method to prioritize the optimization of one of the two criteria (of relevance or redundancy) at the cost of the optimization of the other. Unlike other existing correlation-based techniques, our proposed feature selection techniques do not take for granted that the optimizations of both elements of relevance and redundancy are to have equal priorities in the search for the predictor set [11].

Having introduced the element of differential prioritization, we stress the importance of applying a more appropriate evaluation procedure which gives more realistic estimates of accuracy than the **i**nternal **c**ross **v**alidation (ICV) procedure used in several feature selection studies for gene expression data [3-5]. This is done by evaluating our feature selection techniques using the *F*-splits evaluation procedure.

In this paper, we investigate the efficacy of two DDP-based predictor set scoring methods on nine multiclass microarray datasets. Each of the two methods is differentiated from the other method by the measure of correlations between genes used in the method. The first method is termed the antiredundancy-based *W*_*A,S *_scoring method. The measure of antiredundancy, *U*_*S*_, is used as the measure of correlations between genes in the *W*_*A,S *_scoring method. In the second method, called the redundancy-based *W*_*R,S *_scoring method, the measure of redundancy, *R*_*S*_, is used as the measure of correlations between genes. The DDP parameters for the *W*_*A,S *_and the *W*_*R,S *_scoring methods are denoted as *α *and *ρ *respectively. Larger DDP means more emphasis on optimizing relevance, *V*_*S*_, and less emphasis on minimizing correlations between genes in the predictor set. Conversely, smaller DDP indicates more emphasis on minimizing correlations between members of the predictor set and less emphasis on optimizing its relevance.

The main contribution of this study is to show that a degree of freedom in adjusting the priorities between maximizing relevance and minimizing redundancy is necessary to produce the best classification performance (i.e. equal-priorities techniques might not yield the optimal predictor set). A secondary contribution is to determine which one of the two measures investigated in this study is the better measure of correlations between genes in the predictor set ('predictor genes').

## Results

Nine multiclass microarray datasets are used as benchmark datasets (Table 1). The **Br**ow**n **(BRN) dataset, first analyzed by Munagala *et al*. (2004) [12,13], includes 15 broad cancer types. We also analyzed another version of this dataset, denoted as BRN14, where one class (skin tissue) is excluded due to its small sample size.

The GCM dataset [2,14] contains 14 tumor classes. For the NCI60 dataset [15,16], only 8 tumor classes are analyzed; the 2 samples of the prostate class are excluded due to small class size.

The PDL dataset [17,18] consists of 6 classes, each class representing a diagnostic group of childhood leukemia. The SRBC dataset [19,20] consists of 4 subtypes of small, round, blue cell tumors (SRBCTs). In the 5-class lung dataset [21,22], 4 classes are subtypes of lung cancer; the fifth class consists of normal samples.

The MLL dataset [23,24] contains 3 subtypes of leukemia: ALL, MLL and AML. The AML/ALL dataset [25,26] also contains 3 subtypes of leukemia: AML, B-cell and T-cell ALL.

Except for the BRN, BRN14 and SRBC datasets (which are only available as preprocessed in their originating studies), datasets are preprocessed and normalized based on the recommended procedures in [27] for Affymetrix and cDNA microarray data. Except for the GCM dataset, for which the original ratio of training set size to test set size used in [2] is maintained to enable comparison with previous studies, for all datasets we employ the standard 2:1 split ratio.

Different values of *α *and *ρ *ranging from 0.1 up to 1 with equal intervals of 0.1 are tested. Predictor sets ranging from size *P *= 2 to *P = P*_max _are formed in each split. The number of splits, *F *is set to 10 in this study. The choice of the value of *P*_max _is based on previous studies on feature selection in tumor classification such as [1], where it is observed that there is no significant change in accuracy at values of *P *beyond 150. Therefore, for datasets with larger number of classes (*K*>6), we set *P*_max _to 150, while for datasets with 6 or less classes, the value of *P*_max _is decreased accordingly in proportion to *K *(Table 1). The rationale for this is that datasets with smaller number of classes need less predictor genes to differentiate samples from different classes than datasets with larger number of classes. It is easier to distinguish among say, 3 classes than telling apart samples from 15 different classes (and hence, a smaller predictor set would be sufficient for the former whereas a larger number of predictor genes would be necessary to accomplish the latter).

Two feature selection experiments were run on each dataset: one using the *W*_*A,S *_scoring method and the other, the *W*_*R,S *_scoring method. The DAGSVM classifier is used in evaluating the performance of all resulting predictor sets from both experiments. The DAGSVM is an all-pairs SVM-based multi-classifier which uses substantially less training time compared to either the standard algorithm or Max Wins, and has been shown to produce accuracy comparable to both of these algorithms [28].

**Figure 1:**
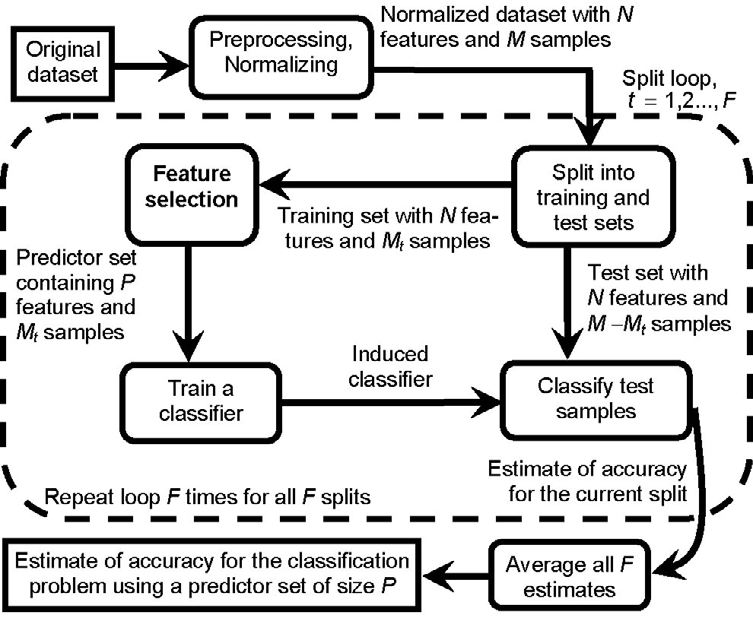
*F*-splits evaluation procedure at each value of *P*.

Two parameters will be used to evaluate the performance of the *W*_*A,S *_and the *W*_*R,S *_scoring methods. The first is the *best estimate of accuracy*. This is simply taken as the largest among the accuracies obtained from Figure 1 at all tested values of *α *or *ρ *at *P = P*_max_. By taking the accuracy at fixed value of *P *(at *P*_max_), we further exclude the possibility of leaking information from the test set into the training process of forming the predictor set in each split. If a draw occurs in terms of the estimate of accuracy, we take the average of the values of *α *or *ρ *giving the largest accuracies as the optimal *α *or *ρ*.

For multiclass classification problems, merely attaining a good estimate of accuracy does not represent excellent classification performance. There is also the need to ensure that samples from all classes are predicted with equally good rate of accuracy. This is especially true when class sizes are greatly unequal among the classes, which is often the case for multiclass microarray datasets. A predictor set may achieve high estimate of overall accuracy by simply predicting test samples as belonging to one of the classes with large class size at a high frequency. The end results will be that samples belonging to certain classes will be correctly predicted most of the time, while samples from other classes will be wrongly classified at a high rate.

This calls for the second parameter, the *range of class accuracies*, in evaluating the performance of the predictor set scoring methods. For each class, class accuracy denotes the ratio of correctly classified samples of that class to the class size in the test set. Each class accuracy is computed from the DDP value which produces the best estimate of accuracy at *P = P*_max _in the first place. The range of class accuracies is the difference between the best class accuracy and the worst class accuracy among the *K *class accuracies in a *K*-class dataset. In an ideal situation, overall accuracy being exactly 1, each class accuracy is 1, so the perfect range of class accuracies is 0. Hence, the lower the range of class accuracies, the better the classification performance.

### Best estimate of accuracy

Overall the *W*_*A,S *_scoring method outperforms the *W*_*R,S *_scoring method by giving better accuracy in six out of nine datasets (Table 2). Only in three datasets, GCM, NCI60 and SRBC datasets, does the *W*_*R,S *_scoring method give the same accuracy as the *W*_*A,S *_scoring method.

**Figure 2:**
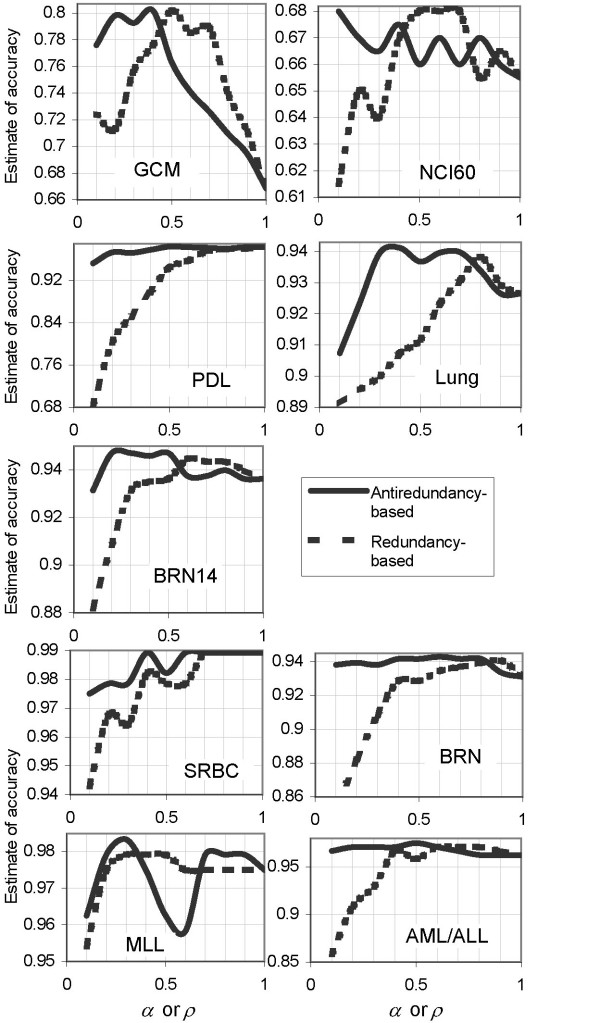
**Estimate of accuracy at *P*=*P*_max _vs. *α *or *ρ***. Solid line: *W*_*A,S *_scoring method, dashed line: *W*_*R,S*_.

Figure 2 shows how the estimate of accuracy at *P *= *P*_max _varies against corresponding value of *α *and *ρ *for the *W*_*A,S *_and the *W*_*R,S *_scoring methods respectively.

### Range of class accuracies

The *W*_*A,S *_scoring method gives better performance than the *W*_*R,S *_scoring method by yielding smaller range of class accuracies for five datasets: GCM, NCI60, PDL, MLL and AML/ALL datasets (Table 3). The *W*_*R,S *_scoring method turns out lower range of class accuracies for only two datasets: the lung and BRN14 datasets. For the remaining two datasets (BRN and SRBC), both methods yield the same performance.

**Figure 3:**
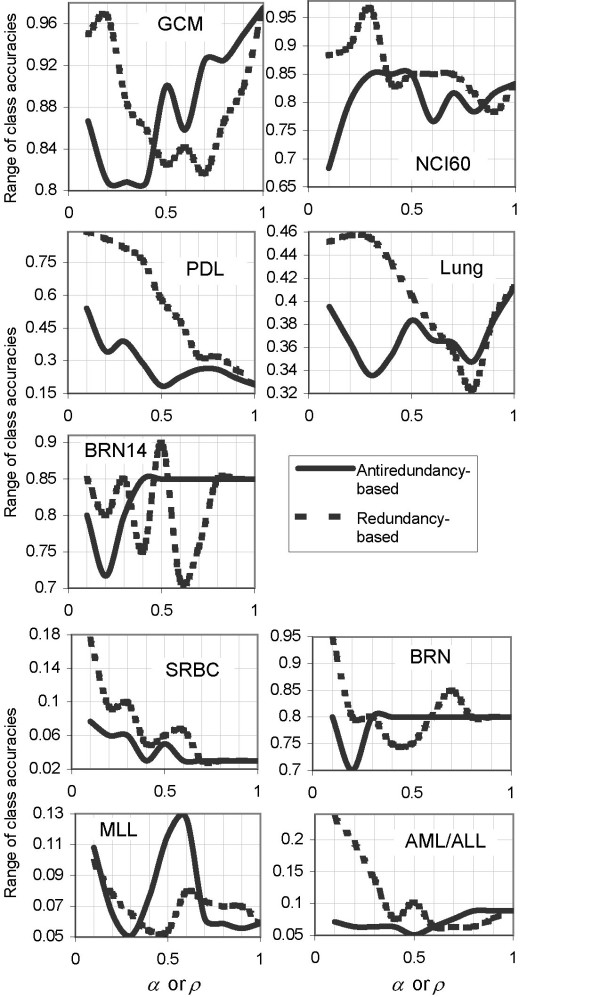
**Range of class accuracies at *P *= *P*_max _vs. *α *or *ρ***. Solid line: *W*_*A,S *_scoring method, dashed line: *W*_*R,S*_.

Figure 3 shows how the range of class accuracies at *P *= *P*_max _varies against corresponding value of *α *and *ρ *for the *W*_*A,S *_and the *W*_*R,S *_scoring methods respectively.

### Comparing the *W_A,S _*and the *W_R,S _*scoring methods

By taking the rightmost columns of Tables 2 and 3, we assign the overall superior method for each of the nine datasets in Table 4. At *P *= *P*_max_, the *W*_*A,S *_scoring method is superior to the *W*_*R,S *_scoring method for six out of nine datasets (15-class BRN, 14-class GCM, 8-class NCI60, 6-class PDL, 3-class MLL and 3-class AML/ALL). Four of these six datasets contain large number of classes (more than 5 classes). The overall superior method is undecided for two datasets (BRN14 and lung datasets), while for the SRBC dataset, both methods produce equal performance in terms of both best estimate of accuracy and range of class accuracies.

Both methods have been briefly compared in a previous work [29] using only one dataset (the GCM dataset). Here we add the binomial test recommended in [30] for comparing classifiers in order to compare both methods for all values of *P *ranging from 2 to *P*_max_. For each predictor set size *P *= 2,3,...,*P*_max_, we identify the DDP (*α *or *ρ*) value which gives the best accuracy (averaged across *F *splits) for each scoring method. In each split, a classifier is constructed using the *P*-gene predictor set obtained at this optimal DDP value from each scoring method. We then compare the two resulting classifiers across splits using the test sets of all *F *splits.

**Figure 4:**
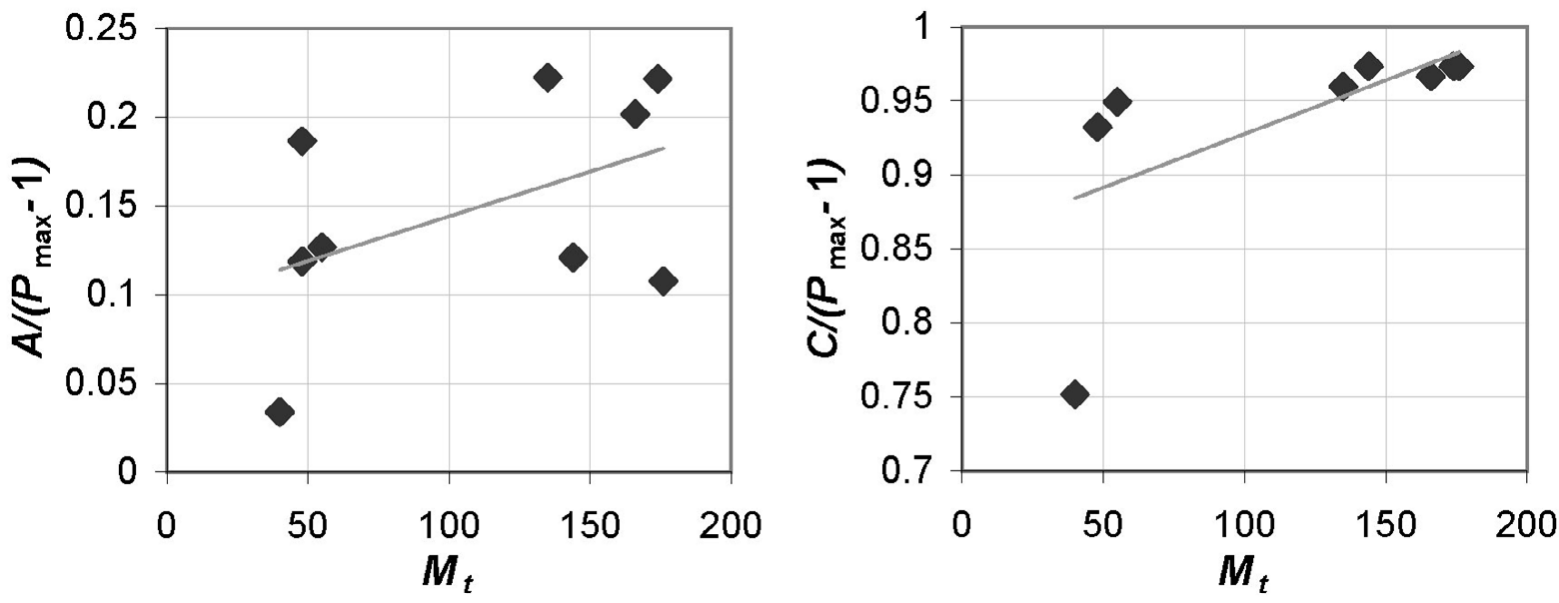
**Correlations of *A *and *C *to training set size, *M*_*t*_**. *A *(left) and *C *(right) plotted against training set size, *M*_*t*_, for all benchmark datasets. *A *and *C *are normalized by dividing against their maximum value, *P*_max_- 1.

Out of these *P*_max_- 1 comparisons, for each dataset we record the number of times, *A*, we reject the null hypothesis that both scoring methods are equal, in favor of the hypothesis that the *W*_*A,S *_scoring method is better than the *W*_*R,S *_scoring method at the 0.05 significance level (Table 5). The outcome of the comparisons does not seem impressive until we take into account the fact that the number of times, *B*, we reject the null hypothesis that both scoring methods are equal, in favor of the hypothesis that the *W*_*A,S *_scoring method is *worse *than the *W*_*R,S *_scoring method, is **0 **for all datasets. Moreover, we observe a strong correlation between the training set size (Table 1) and *A *– the larger the training set, the higher the frequency at which the null hypothesis can be rejected in favor of the *W*_*A,S *_scoring method as the superior method (Figure 4). Therefore, we believe that with sufficiently large training set, it can be irrefutably proven that the *W*_*A,S *_scoring method is the superior method.

To reinforce the results from the binomial test, we further conduct the Wilcoxon signed rank test [31] on accuracies from both methods obtained at the best DDP for each predictor set size *P *= 2,3,...,*P*_max_. The rightmost column of Table 5 contains the number of times, *C*, the right-sided *p*-value is below the significance level of 0.05. The right-sided *p*-value in this case is the probability that *T*_0 _is less than or equal to the Wilcoxon signed rank test statistic, *T*.

T=∑P=2Pmax⁡sign(θP−ηP)⋅Rank(P)     (1)
 MathType@MTEF@5@5@+=feaafiart1ev1aaatCvAUfKttLearuWrP9MDH5MBPbIqV92AaeXatLxBI9gBaebbnrfifHhDYfgasaacH8akY=wiFfYdH8Gipec8Eeeu0xXdbba9frFj0=OqFfea0dXdd9vqai=hGuQ8kuc9pgc9s8qqaq=dirpe0xb9q8qiLsFr0=vr0=vr0dc8meaabaqaciaacaGaaeqabaqabeGadaaakeaacqWGubavcqGH9aqpdaaeWbqaaGqaaiab=nhaZjab=LgaPjab=DgaNjab=5gaUjabcIcaOGGaciab+H7aXnaaBaaaleaacqWGqbauaeqaaOGaeyOeI0Iae43TdG2aaSbaaSqaaiabdcfaqbqabaGccqGGPaqkcqGHflY1cqWFsbGucqWFHbqycqWFUbGBcqWFRbWAcqGGOaakcqWGqbaucqGGPaqkcaWLjaGaaCzcaiabcIcaOiabigdaXiabcMcaPaWcbaGaemiuaaLaeyypa0JaeGOmaidabaGaemiuaa1aaSbaaWqaaiGbc2gaTjabcggaHjabcIha4bqabaaaniabggHiLdaaaa@5637@

where *θ*_*P *_is the number of test samples the *W*_*A,S *_scoring method predicts correctly at predictor set size *P *for all *F *splits, and *η*_*P *_is the number of test samples the *W*_*R,S *_scoring method predicts correctly at predictor set size *P *for all *F *splits. Rank(*P*) is the rank of (*θ*_*P *_- *η*_*P*_). *T*_0 _is computed in a similar way to *T*, except that the expression sign (*θ*_*P *_- *η*_*P*_) is replaced with independent random sign. This means that the right-sided *p*-value represents the probability that the *W*_*A,S *_scoring method is only superior to the *W*_*R,S *_scoring method by *random chance*, given available observations of the classification performance of the two scoring methods. In other words, a right-sided *p*-value that is near zero (less than the 0.05 significance level) indicates a high likelihood that the *W*_*A,S *_scoring method is indeed significantly superior to the *W*_*R,S *_scoring method.

The left-sided *p*-value represents the probability that *T*_0 _is greater than or equal to *T*. Supporting the results from the binomial test, the number of times, *D*, the left-sided *p*-value is below the significance level of 0.05, is also **0 **for all nine datasets. Moreover, as in the case of *A, C *is also proportionate to the training set size, *M*_*t*_, of the corresponding datasets (Figure 4). Indeed, as shown in Figure 4, the ratio of *C *to *P*_max _- 1 has stronger correlation to *M*_*t *_than the ratio of *A *to *P*_max _- 1.

## Discussion

### Comparisons to other studies

Detailed comparisons to previously reported results will only be made for the four datasets with the largest *K*. Two of them, the GCM and NCI60 datasets, have been extensively analyzed in previous studies and have been known to consistently produce low realistic estimates of accuracy (<90%) [1,27,32]. Since the *W*_*A,S *_scoring method has been shown to outperform the *W*_*R,S *_scoring method, we shall compare results from the *W*_*A,S *_scoring method against results from other studies.

For the GCM dataset, with a 150-gene predictor set, an accuracy of 80.2% is achievable with our *W*_*A,S *_scoring method when the value of *α *is set to 0.4. This is a significant improvement compared to the 78% accuracy obtained, using all available 16000 genes, in the original analysis of the same dataset [2]. However, strict comparison cannot be made against this 78% accuracy of [2] and the 81.5% accuracy (using 84 genes) achieved in [32] since the evaluation procedure in both studies is based on a single (the original) split of the dataset. We can make a more appropriate comparison, however, against a comprehensive study on various rank-based feature selection techniques [1]. The study uses external 4-fold cross validation to evaluate classification performance. In [1], the best accuracy for the GCM dataset is 63.3%, when *no *feature selection is applied prior to classification!

For the NCI60 dataset, using the *W*_*A,S *_scoring method, the best 10-splits accuracy of 68% occurs at *α *= 0.1. This is only marginally better than the best accuracy obtained from the two studies employing evaluation procedures similar to ours [1,27]. In [27], the best averaged accuracy is around 67% (using 200 genes selected from the BSS/WSS rank-based feature selection technique), whereas the study by Li *et al*. (2004) [1] gives similar performance with an accuracy of 66.7% achieved using the sum minority rank-based feature selection technique with the same number of genes as our predictor set, 150. A more recent study on a wrapper-based feature selection technique [7] found a LOOCV (**l**eave-**o**ne-**o**ut **c**ross **v**alidation) accuracy of 76.2% for the NCI60 dataset. Unfortunately no separate test set has been formed to validate the efficacy of the 30-gene predictor set which has achieved this accuracy.

The classification of samples from the NCI60 dataset is well-known for being a difficult problem. Major culprits include the small class sizes and the heterogeneity of some of the classes (breast and non-small-cell lung cancer) [27]. Using the current interval size between the DDP values (0.1), our improvement of accuracy for this dataset is small. However, we hope that further refinement to our feature selection technique (through the development of a method to predict the suitable range of the DDP for the dataset of interest, which will enable us to test for both smaller range and interval size for the DDP) will bring about a significantly better accuracy.

The discriminative margin clustering method used on the BRN dataset in [12] is geared towards discovering sub-classes in broad histological types – but manages to yield good class accuracy (70 to 90%) for four tumor classes (kidney, lung, ovary and soft tissue). Not surprisingly, the class accuracy produced by our *W*_*A,S *_scoring method for each of these classes ranges from 90 to 100%. For this dataset, we obtain a 94.3% accuracy using a 150-gene predictor set found at *α *= 0.6.

This is better than the results reported in [6] where an 81.23% LOOCV accuracy on the BRN dataset is achieved using a wrapper-based GA/SVM feature selection technique. However, if the LOOCV accuracy itself is used as the GA fitness function, as is the case in [6], an external test set should have been set aside to evaluate the performance of the technique. It is the accuracy from this test set that provides a realistic estimate of accuracy for the feature selection technique (more details in **The *F*-splits evaluation procedure **sub-section under the **Methods **section). Again, similar to the situation in an aforementioned study on the NCI60 dataset [7], no such evaluation procedure has been implemented in [6].

In [33] the authors have eliminated the skin tissue samples from the 15-class BRN dataset (and thus forming the 14-class BRN14 dataset), also possibly, as in our case, due to small class size (3 samples). In that study, the nearest shrunken centroid classifier yields a 10-splits accuracy of 93.5% using 4327 genes for the BRN14 dataset. This is slightly lower than the 94.7% accuracy from the *W*_*A,S *_scoring method (at *α *= 0.33). More importantly, we use a much smaller predictor set (150 genes) to achieve a better accuracy.

Therefore, compared to previous studies which used realistic evaluation procedures similar to the *F*-splits evaluation procedure, rather than the potentially overly optimistic ICV procedure, our *W*_*A,S *_scoring method has produced better classification performance on highly multiclass datasets such as the BRN, BRN14, GCM and NCI60 datasets.

### The importance of the DDP

In both scoring methods, it is worth noting from Figures 2 and 3, and Table 2 that the best classification performance (i.e. highest accuracy and lowest range of class accuracies) is not always achieved at values of the DDP where the technique becomes equal-priorities scoring method (*α *or *ρ *equals 0.5) or rank-based (*α *or *ρ *equals 1). Therefore, without varying the DDP so that it takes any other values aside from 0.5 or 1, the optimal classification performance would not have been achievable for most of the datasets. For datasets where the optimal value of the DDP happens to be exactly 0.5 (for instance, the PDL dataset where the value of *α *giving the best estimate of accuracy is 0.5), it is due to the fact that some characteristic(s) of the dataset dictate that the optimal value of the DDP for the dataset should be 0.5.

In [11], we have hypothesized that for a given scoring method, the value of the DDP leading to the best estimate of accuracy is dataset-specific. Successfully predicting such optimal value of the DDP for a dataset gives us savings in terms of computational cost and time (we will not have to run feature selection for the full domain of the DDP from 0 to 1). Linking the optimal value of the DDP to dataset characteristic(s) is the first step towards successful prediction of the optimal value of the DDP for any future untested datasets.

**Figure 5:**
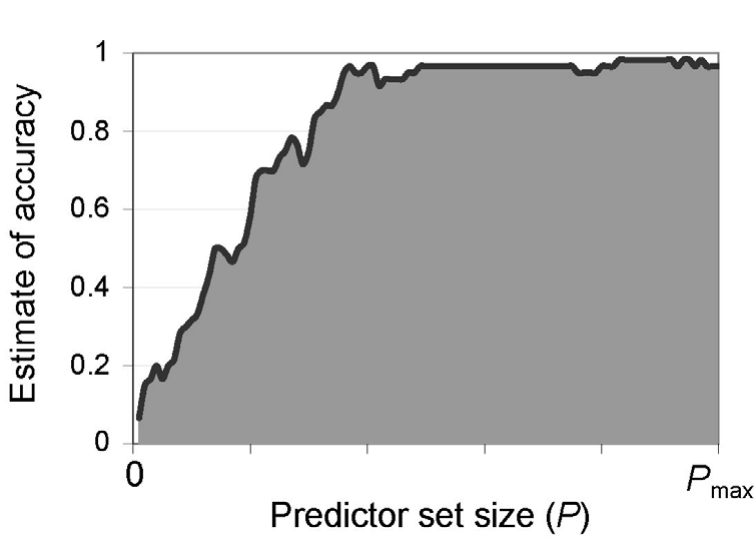
Area under the accuracy-predictor set size curve.

Since our feature selection technique does not explicitly predict the best *P* from the range of [2,* P*_max_], in order to determine the value of the DDP most likely to produce the optimal accuracy, we use a parameter called *size-averaged accuracy*, which is computed as follows. For all predictor sets found using a particular value of the DDP, we plot the estimate of accuracy obtained from the procedure outlined in Figure 1 against the value of *P *of the corresponding predictor set (Figure 5). The size-averaged accuracy for that value of the DDP is the area under the curve in Figure 5 divided by the number of predictor sets, (*P*_max _-1). The value of *α *or *ρ *associated with the highest size-averaged accuracy is deemed the empirical estimate of α* or ρ* (the empirical optimal value of the DDP). If there is a tie in terms of the highest size-averaged accuracy between different values of *α *or *ρ *the empirical estimate of α* or ρ* is taken as the average of those values of *α *or *ρ *[34]

**Figure 6:**
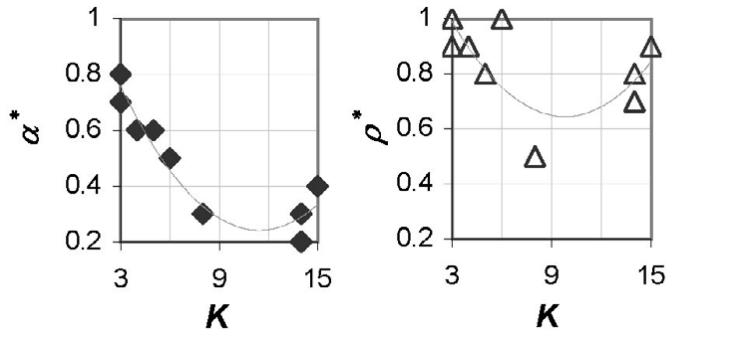
**Relationship between optimal value of the DDP and number of classes in the dataset**. Optimal value of the DDP plotted against number of classes in the dataset for the *W*_*A,S *_scoring method (left) and the *W*_*R,S *_scoring method (right) for all benchmark datasets.

The overall trend in Figure 6 implies that as *K *increases, in order to achieve the optimal classification performance, the emphasis on

• maximizing antiredundancy (for the *W*_*A,S *_scoring method) or

• minimizing redundancy (for the *W*_*R,S *_scoring method)

needs to be increased at the cost of the emphasis on maximizing relevance. Conversely, maximizing antiredundancy (or minimizing redundancy) becomes less important as *K *decreases – thereby supporting the assertion in [9] that redundancy does *not *hinder the discriminant power of the predictor set when *K *is 2. The α* - *K *plot follows this trend more closely than the ρ* - *K *plot.

Since the measure of antiredundancy, *U*_*S*_, and the measure of redundancy, *R*_*S*_, play increasingly important roles compared to relevance, *V*_*S*_, as *K *increases, the better performance of the *W*_*A,S *_scoring method compared to the *W*_*R,S *_scoring method for majority of datasets with larger *K *(> 5) must be due to the superiority of the measure of antiredundancy, *U*_*S*_, over the measure of redundancy, *R*_*S*_, in measuring correlations between predictor genes.

The statement above can be substantiated by comparing the corresponding value of α* to ρ* for each of these datasets in Figure 6. The value of α* is always **less **than the value of ρ* for all datasets. The role of *α *or *ρ *is such that the smaller the value of *α *or *ρ*, the more the emphasis on maximizing *U_S _*or minimizing *R_S_*, respectively (equations 7 and 8). This implies that *U_S _*is more useful than *R_S _*as a criterion in finding the optimal predictor set for datasets with large *K*. Moreover, we observe from Figure 2 that the estimate of accuracy from the *W_R,S _*scoring method at small *ρ *is much lower than accuracy from the *W_A,S _*scoring method at small *α*, again underscoring the reliability of *U_S _over R_S_* in finding the optimal predictor set.

### Most frequently selected genes

Since the *W*_*A,S *_scoring method has been shown to outperform the *W*_*R,S *_scoring method, we perform the analysis on the most frequently selected genes using the optimal predictor sets found from the *W*_*A,S *_scoring method. The optimal predictor sets consist of the *P*_max_-gene predictor set obtained in each split of training and test sets using the value of *α *which gives the best estimate of accuracy (Table 2). The most frequently selected genes are identified by surveying the optimal predictor sets for genes that are selected 10 times out of 10 splits of training and test sets. We then rank these genes based on their split-averaged position in the predictor set. Higher rank is assigned to genes with consistently high position in the predictor set in each split. The biological significance of the top 25 genes is briefly described in Tables 6, 7 and 8 for the BRN, BRN14 and GCM datasets respectively (the three largest datasets in terms of the number of classes).

For the BRN and BRN14 datasets, we first identify which of the 25 genes have been found to be markers for *specific *tumor types against normal tissues in the originating study by Munagala *et al*. [12]. We discover that out of the 25 genes, 10 and 8 genes are included in lists of genes which differentiate specific tumor types from normal tissues in case of the BRN (Table 6) and BRN14 (Table 7) datasets respectively. These lists are available at the website [13] of the authors of [12].

Based on existing literature regarding them [35-48], the remaining 15 (BRN dataset) and 17 (BRN14 dataset) genes can be divided into four groups. The first group is similar to the aforementioned 10 and 8 genes; this group marks a specific cancer class against normal tissues. Thus it is probable that genes which mark a specific tumor type against normal tissues also differentiate that tumor type from all other tumor types.

The second group comprises genes which are known to either promote or inhibit tumor in general (for example, genes #9 and #19 in Table 6; and genes #12 and #15 in Table 7). Our results suggest that these genes are expressed variably among different tumor types as well as between tumor tissues and normal tissues.

The third group contains genes which are tissue-specific (highly expressed in certain tissue relative to other parts of the body). Examples are genes #22, #24 and #25 in Table 6; and genes #22, #23 and #25 in Table 7. This is expected, as the classification problem involves distinguishing among different broad tumor types, each of which originates from a distinct tissue type.

The fourth group is made of unknown sequences and genes with either still-unidentified function (genes #3 and #23 in Table 6; genes #4 and #13 in Table 7) or general housekeeping roles such as production of normal proteins and gene regulation (genes #14 and #18 in Table 6; genes #7 and #21 in Table 7). In other words, these are genes that ostensibly play no role in influencing the development of tumor in general or specific tumor types. However, the identification of these genes as predictor genes for multiclass tumor classification points to the possible cascade effect of these genes in development of specific tumor types, especially in case of gene #18 in Table 6 (also gene #21 in Table 7), which is involved in regulating the expression of other genes.

For the GCM dataset, we also first compare our top 25 genes to the marker genes identified in the originating study by Ramaswamy *et al*. [2]. The authors of [2] have provided a list of OVA (one-vs.-all) marker genes for each tumor type at the paper website [49]. Each list contains the top 1000 genes which distinguish a specific tumor type against all other 13 tumor types in the GCM dataset. These genes are ranked based on their significance, which is computed using the permutation test elucidated in [25]. Out of our top 25 genes, 18 genes (72%) are included in the top 50 of one or more of Ramaswamy *et al*.'s lists of OVA marker genes (Table 8).

Of the remaining 7 genes, only 1 gene is not listed in any of the lists of the top 1000 OVA marker genes of [2]. This is gene #14 in Table 8, which belongs to the second of the four groups of genes defined previously. The other 6 genes belong to the first of the four groups of genes (markers or repressors of specific types of tumor) [50-56].

## Conclusion

For majority of the benchmark datasets, using the optimal value of the degree of differential prioritization gives an accuracy higher than accuracies obtainable using equal-priorities scoring method (*α *or *ρ *fixed at 0.5) or rank-based technique (*α *or *ρ *fixed at 1). Therefore, instead of limiting ourselves to a fixed universal set of priorities for relevance and antiredundancy/redundancy (*α *or *ρ *fixed at 0.5 or 1) for **all **datasets, a suitable range of *α *or *ρ *should be chosen based on the characteristics of the dataset of interest in order to achieve the optimal accuracy.

Furthermore, the study demonstrates the advantages of using the measure of antiredundancy over the measure of redundancy for measuring gene correlations, especially for datasets with large number of classes. Based on the criteria of best estimate of accuracy and range of class accuracies, the antiredundancy-based predictor set scoring method performs better than the redundancy-based predictor set scoring method for majority of the benchmark datasets. Furthermore, the antiredundancy-based predictor set scoring method is the superior method of the two in four of the datasets with the largest number of classes. These are the BRN, PDL, GCM and NCI60 datasets, the last two of which remain the most difficult datasets to work on in the area of tissue classification.

Finally, a large portion of the genes most frequently selected into the optimal predictor sets has been identified by the originating studies on the corresponding datasets as marker genes of specific tumor types. Majority of the most frequently selected genes have also been discovered to be involved in either development or suppression of specific tumor types by other studies. These findings confirm the practical value of our feature selection technique for the analysis of gene expression data.

## Methods

### Terminology and objective

For gene expression datasets, the terms *gene *and *feature *may be used interchangeably. The training set upon which feature selection is to be implemented, T, consists of *N *genes and *M*_*t *_training samples. Sample *j *is represented by a vector, **x**_*j*_, containing the expression of the *N *genes [*x*_1,*j*_,..., *x*_*N,j*_]^T ^and a scalar, *y*_*j*_, representing the class the sample belongs to. The target class vector **y **is defined as [*y*_1_, ..., *y*_*Mt*_], *y*_*j *_∈[1, *K*] in a *K*-class dataset. From the total of *N *genes, the objective is to form the subset of genes, called the predictor set *S*, which gives the optimal classification accuracy.

### Two predictor set scoring methods

A score of goodness incorporating both the elements of maximum relevance and minimum redundancy ensures that the predictor set should possess maximal power in discriminating among different classes (maximum relevance), while at the same time containing features with minimal correlation to each other (minimum redundancy).

The relevance of a predictor set *S *is the average of score of relevance, *F*(*i*) of all members of *S*, as recommended in [4]:

VS=1|S|∑i∈SF(i)     (2)
 MathType@MTEF@5@5@+=feaafiart1ev1aaatCvAUfKttLearuWrP9MDH5MBPbIqV92AaeXatLxBI9gBaebbnrfifHhDYfgasaacH8akY=wiFfYdH8Gipec8Eeeu0xXdbba9frFj0=OqFfea0dXdd9vqai=hGuQ8kuc9pgc9s8qqaq=dirpe0xb9q8qiLsFr0=vr0=vr0dc8meaabaqaciaacaGaaeqabaqabeGadaaakeaacqWGwbGvdaWgaaWcbaGaem4uamfabeaakiabg2da9maalaaabaGaeGymaedabaWaaqWaaeaacqWGtbWuaiaawEa7caGLiWoaaaWaaabuaeaacqWGgbGrcqGGOaakcqWGPbqAcqGGPaqkaSqaaiabdMgaPjabgIGiolabdofatbqab0GaeyyeIuoakiaaxMaacaWLjaGaeiikaGIaeGOmaiJaeiykaKcaaa@43E2@

*F*(*i*) is the score of relevance for gene *i*. It indicates the correlation of gene *i *to the target class vector **y**. A popular parameter for computing *F*(*i*) is the BSS/WSS (**b**etween-groups **s**um of **s**quares/**w**ithin-groups **s**um of **s**quares) ratio used in [4,27]. For gene *i*,

F(i)=∑j=1Mt∑k=1KI(yj=k)(x¯ik−x¯i·)2∑j=1Mt∑k=1KI(yj=k)(xij−x¯ik)2     (3)
 MathType@MTEF@5@5@+=feaafiart1ev1aaatCvAUfKttLearuWrP9MDH5MBPbIqV92AaeXatLxBI9gBaebbnrfifHhDYfgasaacH8akY=wiFfYdH8Gipec8Eeeu0xXdbba9frFj0=OqFfea0dXdd9vqai=hGuQ8kuc9pgc9s8qqaq=dirpe0xb9q8qiLsFr0=vr0=vr0dc8meaabaqaciaacaGaaeqabaqabeGadaaakeaacqWGgbGrcqGGOaakcqWGPbqAcqGGPaqkcqGH9aqpdaWcaaqaamaaqahabaWaaabCaeaacqWGjbqscqGGOaakcqWG5bqEdaWgaaWcbaGaemOAaOgabeaakiabg2da9iabdUgaRjabcMcaPiabcIcaOiqbdIha4zaaraWaaSbaaSqaaiabdMgaPjabdUgaRbqabaGccqGHsislcuWG4baEgaqeamaaBaaaleaacqWGPbqAcqWIpM+zaeqaaOGaeiykaKYaaWbaaSqabeaacqaIYaGmaaaabaGaem4AaSMaeyypa0JaeGymaedabaGaem4saSeaniabggHiLdaaleaacqWGQbGAcqGH9aqpcqaIXaqmaeaacqWGnbqtdaWgaaadbaGaemiDaqhabeaaa0GaeyyeIuoaaOqaamaaqahabaWaaabCaeaacqWGjbqscqGGOaakcqWG5bqEdaWgaaWcbaGaemOAaOgabeaakiabg2da9iabdUgaRjabcMcaPiabcIcaOiabdIha4naaBaaaleaacqWGPbqAcqWGQbGAaeqaaOGaeyOeI0IafmiEaGNbaebadaWgaaWcbaGaemyAaKMaem4AaSgabeaakiabcMcaPmaaCaaaleqabaGaeGOmaidaaaqaaiabdUgaRjabg2da9iabigdaXaqaaiabdUealbqdcqGHris5aaWcbaGaemOAaOMaeyypa0JaeGymaedabaGaemyta00aaSbaaWqaaiabdsha0bqabaaaniabggHiLdaaaOGaaCzcaiaaxMaacqGGOaakcqaIZaWmcqGGPaqkaaa@7EDB@

where *I*(.) is an indicator function returning 1 if the condition inside the parentheses is true, otherwise it returns 0. x¯i·
 MathType@MTEF@5@5@+=feaafiart1ev1aaatCvAUfKttLearuWrP9MDH5MBPbIqV92AaeXatLxBI9gBaebbnrfifHhDYfgasaacH8akY=wiFfYdH8Gipec8Eeeu0xXdbba9frFj0=OqFfea0dXdd9vqai=hGuQ8kuc9pgc9s8qqaq=dirpe0xb9q8qiLsFr0=vr0=vr0dc8meaabaqaciaacaGaaeqabaqabeGadaaakeaacuWG4baEgaqeamaaBaaaleaacqWGPbqAcqWIpM+zaeqaaaaa@3234@ is the average of the expression of gene *i *across all training samples, while x¯ik
 MathType@MTEF@5@5@+=feaafiart1ev1aaatCvAUfKttLearuWrP9MDH5MBPbIqV92AaeXatLxBI9gBaebbnrfifHhDYfgasaacH8akY=wiFfYdH8Gipec8Eeeu0xXdbba9frFj0=OqFfea0dXdd9vqai=hGuQ8kuc9pgc9s8qqaq=dirpe0xb9q8qiLsFr0=vr0=vr0dc8meaabaqaciaacaGaaeqabaqabeGadaaakeaacuWG4baEgaqeamaaBaaaleaacqWGPbqAcqWGRbWAaeqaaaaa@3123@ is the average of the expression of gene *i *across training samples belonging to class *k*. The BSS/WSS ratio, first used in [27] for multiclass tumor classification, is a modification of the *F*-ratio statistics for one-way ANOVA (Analysis of Variance). It indicates the gene's ability in discriminating among samples belonging to the *K *different classes.

To measure the correlation between genes *i *and *j*, the absolute value of the Pearson product moment correlation coefficient between the two, |*R*(*i,j*)|, is used. Absolute value is used because both extreme correlation and anti-correlation indicates strong similarity between a pair of genes. There are two possible schemes for measuring the correlations among the members of *S*, each leading to a different predictor set scoring method. The first scheme uses direct redundancy. Redundancy is defined as the sum of all possible pairwise correlations in *S *and has been introduced in [4] for use with gene expression datasets.

RS=1|S|2∑i,j∈S,i≠j|R(i,j)|     (4)
 MathType@MTEF@5@5@+=feaafiart1ev1aaatCvAUfKttLearuWrP9MDH5MBPbIqV92AaeXatLxBI9gBaebbnrfifHhDYfgasaacH8akY=wiFfYdH8Gipec8Eeeu0xXdbba9frFj0=OqFfea0dXdd9vqai=hGuQ8kuc9pgc9s8qqaq=dirpe0xb9q8qiLsFr0=vr0=vr0dc8meaabaqaciaacaGaaeqabaqabeGadaaakeaacqWGsbGudaWgaaWcbaGaem4uamfabeaakiabg2da9maalaaabaGaeGymaedabaWaaqWaaeaacqWGtbWuaiaawEa7caGLiWoadaahaaWcbeqaaiabikdaYaaaaaGcdaaeqbqaamaaemaabaGaemOuaiLaeiikaGIaemyAaKMaeiilaWIaemOAaOMaeiykaKcacaGLhWUaayjcSdaaleaacqWGPbqAcqGGSaalcqWGQbGAcqGHiiIZcqWGtbWucqGGSaalcqWGPbqAcqGHGjsUcqWGQbGAaeqaniabggHiLdGccaWLjaGaaCzcaiabcIcaOiabisda0iabcMcaPaaa@521A@

Theoretically the largest possible value of *R*_*S *_is

RS,max⁡=|S|−12|S|     (5)
 MathType@MTEF@5@5@+=feaafiart1ev1aaatCvAUfKttLearuWrP9MDH5MBPbIqV92AaeXatLxBI9gBaebbnrfifHhDYfgasaacH8akY=wiFfYdH8Gipec8Eeeu0xXdbba9frFj0=OqFfea0dXdd9vqai=hGuQ8kuc9pgc9s8qqaq=dirpe0xb9q8qiLsFr0=vr0=vr0dc8meaabaqaciaacaGaaeqabaqabeGadaaakeaacqWGsbGudaWgaaWcbaGaem4uamLaeiilaWIagiyBa0MaeiyyaeMaeiiEaGhabeaakiabg2da9maalaaabaWaaqWaaeaacqWGtbWuaiaawEa7caGLiWoacqGHsislcqaIXaqmaeaacqaIYaGmdaabdaqaaiabdofatbGaay5bSlaawIa7aaaacaWLjaGaaCzcaiabcIcaOiabiwda1iabcMcaPaaa@44B9@

which occurs when every pair of genes in *S *has perfect correlation or anti-correlation (where |*R*(*i,j*)| equals 1). Conversely, the smallest possible value of *R*_*S *_is zero, when every pair of genes in *S *has zero correlation (where |*R*(*i,j*)| equals 0). As |*S*| approaches infinity, the limit of *R*_*S,max *_is 0.5. Hence *R*_*S *_has theoretical bounds of [0,0.5).

The second scheme uses a measure called *antiredundancy *which we proposed in [57]. It quantifies the *lack of redundancy *in *S*.

US=1|S|2∑i,j∈S,i≠j1−|R(i,j)|     (6)
 MathType@MTEF@5@5@+=feaafiart1ev1aaatCvAUfKttLearuWrP9MDH5MBPbIqV92AaeXatLxBI9gBaebbnrfifHhDYfgasaacH8akY=wiFfYdH8Gipec8Eeeu0xXdbba9frFj0=OqFfea0dXdd9vqai=hGuQ8kuc9pgc9s8qqaq=dirpe0xb9q8qiLsFr0=vr0=vr0dc8meaabaqaciaacaGaaeqabaqabeGadaaakeaacqWGvbqvdaWgaaWcbaGaem4uamfabeaakiabg2da9maalaaabaGaeGymaedabaWaaqWaaeaacqWGtbWuaiaawEa7caGLiWoadaahaaWcbeqaaiabikdaYaaaaaGcdaaeqbqaaiabigdaXiabgkHiTmaaemaabaGaemOuaiLaeiikaGIaemyAaKMaeiilaWIaemOAaOMaeiykaKcacaGLhWUaayjcSdaaleaacqWGPbqAcqGGSaalcqWGQbGAcqGHiiIZcqWGtbWucqGGSaalcqWGPbqAcqGHGjsUcqWGQbGAaeqaniabggHiLdGccaWLjaGaaCzcaiabcIcaOiabiAda2iabcMcaPaaa@5401@

With *R*_*S*_, we have a redundancy-based scoring method in which the measure of goodness for predictor set *S *is given as follows.

WR,S=(VS)ρ(RS)1−ρ     (7)
 MathType@MTEF@5@5@+=feaafiart1ev1aaatCvAUfKttLearuWrP9MDH5MBPbIqV92AaeXatLxBI9gBaebbnrfifHhDYfgasaacH8akY=wiFfYdH8Gipec8Eeeu0xXdbba9frFj0=OqFfea0dXdd9vqai=hGuQ8kuc9pgc9s8qqaq=dirpe0xb9q8qiLsFr0=vr0=vr0dc8meaabaqaciaacaGaaeqabaqabeGadaaakeaacqWGxbWvdaWgaaWcbaGaemOuaiLaeiilaWIaem4uamfabeaakiabg2da9maalaaabaGaeiikaGIaemOvay1aaSbaaSqaaiabdofatbqabaGccqGGPaqkdaahaaWcbeqaaGGaciab=f8aYbaaaOqaaiabcIcaOiabdkfasnaaBaaaleaacqWGtbWuaeqaaOGaeiykaKYaaWbaaSqabeaacqaIXaqmcqGHsislcqWFbpGCaaaaaOGaaCzcaiaaxMaacqGGOaakcqaI3aWncqGGPaqkaaa@44BA@

where the power factor *ρ* ∈ (0, 1] denotes the DDP between maximizing relevance and minimizing redundancy [29].

With *U*_*S*_, we have an antiredundancy-based scoring method in which the score of goodness for predictor set *S *is given as follows.

*W*_*A,S *_= (*V*_*S*_)^*α*^·(*U*_*S*_)^1-*α *^    (8)

where the power factor *α* ∈ (0, 1] denotes the DDP between maximizing relevance and maximizing antiredundancy [11]. Codes for both scoring methods are available as MATLAB M-files [see Additional file 1].

### Significance of the DDP

In the previous section it has been stated that a predictor set is to be found based on two criteria: maximum relevance and **either **minimum redundancy or maximum antiredundancy. However, the quantification of the priority to be assigned to each of these two criteria remains an unexplored area.

In the antiredundancy-based scoring method, decreasing the value of *α *forces the search method to put more priority on maximizing antiredundancy at the cost of maximizing relevance. Raising the value of *α *increases the emphasis on maximizing relevance (and at the same time decreases the emphasis on maximizing antiredundancy) during the search for the predictor set.

A predictor set found using larger value of *α *has more features with strong relevance to the target class vector, but also more redundancy among these features. Conversely, a predictor set obtained using smaller value of *α *contains less redundancy among its member features, but at the same time also has fewer features with strong relevance to the target class vector. At *α *= 0.5, we get an equal-priorities scoring method.

*W*_*A,S *_= (*V*_*S*_·*U*_*S*_)^0.5 ^    (9)

At *α *= 1, the feature selection technique becomes rank-based.

*W*_*A,S *_= *V*_*S *_    (10)

There is also the trivial case of *α *= 0, where only antiredundancy is considered in forming the predictor set.

*W*_*A,S *_= *U*_*S *_    (11)

The role and significance of *ρ *in the redundancy-based scoring method are similar to those of *α *in the antiredundancy-based scoring method, which has been elucidated in the paragraphs above. At *ρ *= 0.5, the redundancy-based scoring method is effectively the same as one of the scoring methods presented in [4].

WR,S=(VSRS)0.5     (12)
 MathType@MTEF@5@5@+=feaafiart1ev1aaatCvAUfKttLearuWrP9MDH5MBPbIqV92AaeXatLxBI9gBaebbnrfifHhDYfgasaacH8akY=wiFfYdH8Gipec8Eeeu0xXdbba9frFj0=OqFfea0dXdd9vqai=hGuQ8kuc9pgc9s8qqaq=dirpe0xb9q8qiLsFr0=vr0=vr0dc8meaabaqaciaacaGaaeqabaqabeGadaaakeaacqWGxbWvdaWgaaWcbaGaemOuaiLaeiilaWIaem4uamfabeaakiabg2da9maabmaabaWaaSaaaeaacqWGwbGvdaWgaaWcbaGaem4uamfabeaaaOqaaiabdkfasnaaBaaaleaacqWGtbWuaeqaaaaaaOGaayjkaiaawMcaamaaCaaaleqabaGaeGimaaJaeiOla4IaeGynaudaaOGaaCzcaiaaxMaacqGGOaakcqaIXaqmcqaIYaGmcqGGPaqkaaa@40F9@

The case of *ρ *= 1 is similar to *α *= 1.

*W*_*R,S *_= *V*_*S *_    (13)

In the trivial case of *ρ *= 0, only redundancy is considered during feature selection.

WR,S=1RS     (14)
 MathType@MTEF@5@5@+=feaafiart1ev1aaatCvAUfKttLearuWrP9MDH5MBPbIqV92AaeXatLxBI9gBaebbnrfifHhDYfgasaacH8akY=wiFfYdH8Gipec8Eeeu0xXdbba9frFj0=OqFfea0dXdd9vqai=hGuQ8kuc9pgc9s8qqaq=dirpe0xb9q8qiLsFr0=vr0=vr0dc8meaabaqaciaacaGaaeqabaqabeGadaaakeaacqWGxbWvdaWgaaWcbaGaemOuaiLaeiilaWIaem4uamfabeaakiabg2da9maalaaabaGaeGymaedabaGaemOuai1aaSbaaSqaaiabdofatbqabaaaaOGaaCzcaiaaxMaacqGGOaakcqaIXaqmcqaI0aancqGGPaqkaaa@3AC9@

We posit that different datasets will require different values of DDP between maximizing relevance and minimizing redundancy/maximizing antiredundancy in order to come up with the most efficacious predictor set. Therefore the optimal range of *α *or *ρ *(optimal as in leading to the predictor set giving the best estimate of accuracy) is dataset-specific.

### Search method

For predictor set search, the linear incremental search method [4,5] is used, where the first member of *S *is chosen by selecting the gene with the highest *F*(*i*) score. To find the second and the subsequent members of the predictor set, the remaining genes are screened one by one for the gene that would give the maximum *W*_*A,S *_or *W*_*R,S*_. The procedure is terminated when *P *has reached *P*_max_. *P*_max _is the size of the largest predictor set we wish to look for. This search method has a computational complexity of O(*NP*_max_), which is much lower than that of exhaustive search, O(NPmax⁡
 MathType@MTEF@5@5@+=feaafiart1ev1aaatCvAUfKttLearuWrP9MDH5MBPbIqV92AaeXatLxBI9gBaebbnrfifHhDYfgasaacH8akY=wiFfYdH8Gipec8Eeeu0xXdbba9frFj0=OqFfea0dXdd9vqai=hGuQ8kuc9pgc9s8qqaq=dirpe0xb9q8qiLsFr0=vr0=vr0dc8meaabaqaciaacaGaaeqabaqabeGadaaakeaacqWGobGtdaahaaWcbeqaaiabdcfaqnaaBaaameaacyGGTbqBcqGGHbqycqGG4baEaeqaaaaaaaa@337A@).

### Differences of the roles played by antiredundancy, *U_S_*, and redundancy, *R_S_*

Substituting equation (4) into equation (6), we express the relationship between antiredundancy, *U*_*S*_, and redundancy, *R*_*S*_, as follows.

*U*_*S *_*= χ *- *R*_*S *_⇔ *R*_*S *_= *χ* - *U*_*S *_    (15)

where χ=|S|−12|S|
 MathType@MTEF@5@5@+=feaafiart1ev1aaatCvAUfKttLearuWrP9MDH5MBPbIqV92AaeXatLxBI9gBaebbnrfifHhDYfgasaacH8akY=wiFfYdH8Gipec8Eeeu0xXdbba9frFj0=OqFfea0dXdd9vqai=hGuQ8kuc9pgc9s8qqaq=dirpe0xb9q8qiLsFr0=vr0=vr0dc8meaabaqaciaacaGaaeqabaqabeGadaaakeaacqaHhpWycqGH9aqpdaWcaaqaamaaemaabaGaem4uamfacaGLhWUaayjcSdGaeyOeI0IaeGymaedabaGaeGOmaiZaaqWaaeaacqWGtbWuaiaawEa7caGLiWoaaaaaaa@3AEA@, which, as indicated in equation (5), is also the largest possible value of *R*_*S*_. As |*S*| approaches infinity, the limit of *χ* is 0.5. Therefore, similar to *R*_*S*_, the bounds of *U*_*S *_is also [0,0.5).

The mathematical difference between the *W*_*A,S *_scoring method and the *W*_*R,S *_scoring method contrasts the manner the sum of the absolute value of the Pearson product moment correlation coefficient between all possible pairwise combinations in the predictor set *S*, ∑i,j∈S,i≠j|R(i,j)|
 MathType@MTEF@5@5@+=feaafiart1ev1aaatCvAUfKttLearuWrP9MDH5MBPbIqV92AaeXatLxBI9gBaebbnrfifHhDYfgasaacH8akY=wiFfYdH8Gipec8Eeeu0xXdbba9frFj0=OqFfea0dXdd9vqai=hGuQ8kuc9pgc9s8qqaq=dirpe0xb9q8qiLsFr0=vr0=vr0dc8meaabaqaciaacaGaaeqabaqabeGadaaakeaadaaeqbqaamaaemaabaGaemOuaiLaeiikaGIaemyAaKMaeiilaWIaemOAaOMaeiykaKcacaGLhWUaayjcSdaaleaacqWGPbqAcqGGSaalcqWGQbGAcqGHiiIZcqWGtbWucqGGSaalcqWGPbqAcqGHGjsUcqWGQbGAaeqaniabggHiLdaaaa@4412@, is minimized between the two scoring methods.

In the *W*_*R,S *_scoring method we directly minimize ∑i,j∈S,i≠j|R(i,j)|
 MathType@MTEF@5@5@+=feaafiart1ev1aaatCvAUfKttLearuWrP9MDH5MBPbIqV92AaeXatLxBI9gBaebbnrfifHhDYfgasaacH8akY=wiFfYdH8Gipec8Eeeu0xXdbba9frFj0=OqFfea0dXdd9vqai=hGuQ8kuc9pgc9s8qqaq=dirpe0xb9q8qiLsFr0=vr0=vr0dc8meaabaqaciaacaGaaeqabaqabeGadaaakeaadaaeqbqaamaaemaabaGaemOuaiLaeiikaGIaemyAaKMaeiilaWIaemOAaOMaeiykaKcacaGLhWUaayjcSdaaleaacqWGPbqAcqGGSaalcqWGQbGAcqGHiiIZcqWGtbWucqGGSaalcqWGPbqAcqGHGjsUcqWGQbGAaeqaniabggHiLdaaaa@4412@ by making it a part of the denominator in equation (7). In the *W*_*A,S *_scoring method the minimization is done *indirectly*, where we maximize - [1|S|2∑i,j∈S,i≠j|R(i,j)|]
 MathType@MTEF@5@5@+=feaafiart1ev1aaatCvAUfKttLearuWrP9MDH5MBPbIqV92AaeXatLxBI9gBaebbnrfifHhDYfgasaacH8akY=wiFfYdH8Gipec8Eeeu0xXdbba9frFj0=OqFfea0dXdd9vqai=hGuQ8kuc9pgc9s8qqaq=dirpe0xb9q8qiLsFr0=vr0=vr0dc8meaabaqaciaacaGaaeqabaqabeGadaaakeaadaWadaqaamaalaaabaGaeGymaedabaWaaqWaaeaacqWGtbWuaiaawEa7caGLiWoadaahaaWcbeqaaiabikdaYaaaaaGcdaaeqbqaamaaemaabaGaemOuaiLaeiikaGIaemyAaKMaeiilaWIaemOAaOMaeiykaKcacaGLhWUaayjcSdaaleaacqWGPbqAcqGGSaalcqWGQbGAcqGHiiIZcqWGtbWucqGGSaalcqWGPbqAcqGHGjsUcqWGQbGAaeqaniabggHiLdaakiaawUfacaGLDbaaaaa@4C88@ (note the minus sign) by making *W*_*A,S *_the product of *V*_*S *_and *U*_*S *_in equation (8).

This difference might be a key to the reason the *W*_*A,S *_scoring method works better than the *W*_*R,S *_scoring method. Since the minimum limit of *R*_*S *_is 0, it is possible in practical situations to encounter cases where *R*_*S *_approaches zero. When this occurs, the influence of the denominator in equation (7) greatly overwhelms the contribution of the numerator. This is true even for a large part of the domain of *ρ *(i.e. at least from 0 to 0.6).

Therefore, rather than the bounds of *R*_*S *_itself, the concern is on the bounds of the expression (1RS)1−ρ
 MathType@MTEF@5@5@+=feaafiart1ev1aaatCvAUfKttLearuWrP9MDH5MBPbIqV92AaeXatLxBI9gBaebbnrfifHhDYfgasaacH8akY=wiFfYdH8Gipec8Eeeu0xXdbba9frFj0=OqFfea0dXdd9vqai=hGuQ8kuc9pgc9s8qqaq=dirpe0xb9q8qiLsFr0=vr0=vr0dc8meaabaqaciaacaGaaeqabaqabeGadaaakeaadaqadaqaamaalaaabaGaeGymaedabaGaemOuai1aaSbaaSqaaiabdofatbqabaaaaaGccaGLOaGaayzkaaWaaWbaaSqabeaacqaIXaqmcqGHsisliiGacqWFbpGCaaaaaa@3598@ in equation (7) which is (2,∞ ] for all values of *ρ ∈ *(0,1]. The upper limit leads to instability when redundancy is minimal (near zero), causing the search process to over-emphasize *R*_*S *_at the cost of *V*_*S *_even at larger values of *ρ*. On the other hand, the expression (*U*_*S*_)^1-*α *^in equation (8) has stable limits of (0,1] for all values of *α ∈ *(0,1].

**Figure 7:**
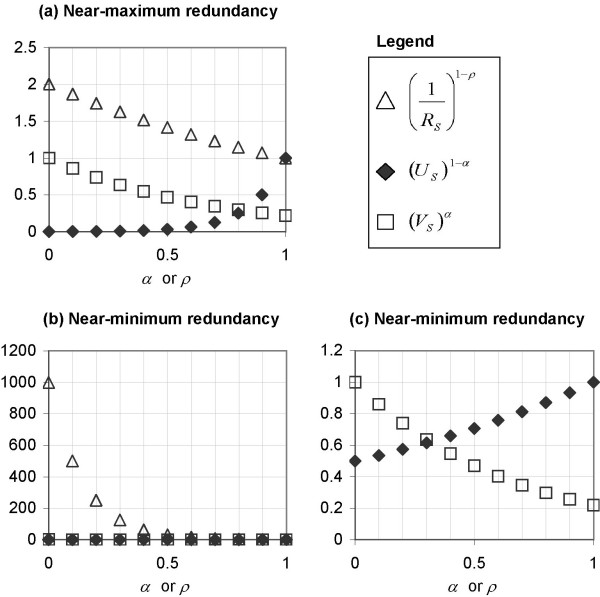
**Limits of (*U*_*S*_)^1-*α *^and **(1RS)1−ρ
 MathType@MTEF@5@5@+=feaafiart1ev1aaatCvAUfKttLearuWrP9MDH5MBPbIqV92AaeXatLxBI9gBaebbnrfifHhDYfgasaacH8akY=wiFfYdH8Gipec8Eeeu0xXdbba9frFj0=OqFfea0dXdd9vqai=hGuQ8kuc9pgc9s8qqaq=dirpe0xb9q8qiLsFr0=vr0=vr0dc8meaabaqaciaacaGaaeqabaqabeGadaaakeaadaqadaqaamaalaaabaGaeGymaedabaGaemOuai1aaSbaaSqaaiabdofatbqabaaaaaGccaGLOaGaayzkaaWaaWbaaSqabeaacqaIXaqmcqGHsisliiGacqWFbpGCaaaaaa@3598@**in extreme situations of near-maximum and near-minimum redundancy**. (*U*_*S*_)^1-*α *^and (1RS)1−ρ
 MathType@MTEF@5@5@+=feaafiart1ev1aaatCvAUfKttLearuWrP9MDH5MBPbIqV92AaeXatLxBI9gBaebbnrfifHhDYfgasaacH8akY=wiFfYdH8Gipec8Eeeu0xXdbba9frFj0=OqFfea0dXdd9vqai=hGuQ8kuc9pgc9s8qqaq=dirpe0xb9q8qiLsFr0=vr0=vr0dc8meaabaqaciaacaGaaeqabaqabeGadaaakeaadaqadaqaamaalaaabaGaeGymaedabaGaemOuai1aaSbaaSqaaiabdofatbqabaaaaaGccaGLOaGaayzkaaWaaWbaaSqabeaacqaIXaqmcqGHsisliiGacqWFbpGCaaaaaa@3598@ plotted against *α *and *ρ *respectively and (*V*_*S*_)^*α *^plotted against the DDP in the cases where (a) redundancy is close to the theoretical maximum, and (b) redundancy is close to the theoretical minimum, and (c) magnification of plot (b) for (*U*_*S*_)^1-*α *^and (*V*_*S*_)^*α*^.

In Figure 7, we plot (*U*_*S*_)^1-*α *^and (1RS)1−ρ
 MathType@MTEF@5@5@+=feaafiart1ev1aaatCvAUfKttLearuWrP9MDH5MBPbIqV92AaeXatLxBI9gBaebbnrfifHhDYfgasaacH8akY=wiFfYdH8Gipec8Eeeu0xXdbba9frFj0=OqFfea0dXdd9vqai=hGuQ8kuc9pgc9s8qqaq=dirpe0xb9q8qiLsFr0=vr0=vr0dc8meaabaqaciaacaGaaeqabaqabeGadaaakeaadaqadaqaamaalaaabaGaeGymaedabaGaemOuai1aaSbaaSqaaiabdofatbqabaaaaaGccaGLOaGaayzkaaWaaWbaaSqabeaacqaIXaqmcqGHsisliiGacqWFbpGCaaaaaa@3598@ against *α *and *ρ *respectively, along with the (*V*_*S*_)*^α ^*DDP plot in order to demonstrate the different ways (*U*_*S*_)^1-*α *^and (1RS)1−ρ
 MathType@MTEF@5@5@+=feaafiart1ev1aaatCvAUfKttLearuWrP9MDH5MBPbIqV92AaeXatLxBI9gBaebbnrfifHhDYfgasaacH8akY=wiFfYdH8Gipec8Eeeu0xXdbba9frFj0=OqFfea0dXdd9vqai=hGuQ8kuc9pgc9s8qqaq=dirpe0xb9q8qiLsFr0=vr0=vr0dc8meaabaqaciaacaGaaeqabaqabeGadaaakeaadaqadaqaamaalaaabaGaeGymaedabaGaemOuai1aaSbaaSqaaiabdofatbqabaaaaaGccaGLOaGaayzkaaWaaWbaaSqabeaacqaIXaqmcqGHsisliiGacqWFbpGCaaaaaa@3598@ change with respect to both the values of the DDP and the two extreme situations of near-maximum redundancy and near-minimum redundancy. For *V*_*S *_we use a typical value of 0.22 (based on the split-averaged mean of the values of *F*(*i*) for the top 150 rank-based genes of the GCM dataset) in both situations.

Figure 7(a) depicts the situation of near-maximum redundancy, where *R*_*S *_approaches 0.5 and accordingly, *U*_*S *_approaches 0. In this case we use the values *R*_*S *_= 0.499 and thus, based on equation (15), *U*_*S *_= 0.001. When the DDP is low (less than 0.7), (*U*_*S*_)^1-*α *^is less than 0.1, and thus it drags the value of *W*_*A,S *_down regardless of the value of *V*_*S *_since *S *is a predictor set with near-maximum redundancy. The aforementioned drag weakens as *α *increases and thus moving away the emphasis from antiredundancy to relevance. (1RS)1−ρ
 MathType@MTEF@5@5@+=feaafiart1ev1aaatCvAUfKttLearuWrP9MDH5MBPbIqV92AaeXatLxBI9gBaebbnrfifHhDYfgasaacH8akY=wiFfYdH8Gipec8Eeeu0xXdbba9frFj0=OqFfea0dXdd9vqai=hGuQ8kuc9pgc9s8qqaq=dirpe0xb9q8qiLsFr0=vr0=vr0dc8meaabaqaciaacaGaaeqabaqabeGadaaakeaadaqadaqaamaalaaabaGaeGymaedabaGaemOuai1aaSbaaSqaaiabdofatbqabaaaaaGccaGLOaGaayzkaaWaaWbaaSqabeaacqaIXaqmcqGHsisliiGacqWFbpGCaaaaaa@3598@ is near 2 when *ρ *is 0 and contributes that value to *W*_*R,S *_; the contribution of (1RS)1−ρ
 MathType@MTEF@5@5@+=feaafiart1ev1aaatCvAUfKttLearuWrP9MDH5MBPbIqV92AaeXatLxBI9gBaebbnrfifHhDYfgasaacH8akY=wiFfYdH8Gipec8Eeeu0xXdbba9frFj0=OqFfea0dXdd9vqai=hGuQ8kuc9pgc9s8qqaq=dirpe0xb9q8qiLsFr0=vr0=vr0dc8meaabaqaciaacaGaaeqabaqabeGadaaakeaadaqadaqaamaalaaabaGaeGymaedabaGaemOuai1aaSbaaSqaaiabdofatbqabaaaaaGccaGLOaGaayzkaaWaaWbaaSqabeaacqaIXaqmcqGHsisliiGacqWFbpGCaaaaaa@3598@ decreases as *ρ *increases, as explained in the previous section on the significance of the DDP. Thus there is no problem with the behaviour of both parameters in the situation where *S *has near-maximum redundancy.

Figure 7(b) shows the plots in situation where *S *has near-minimum redundancy, where *R*_*S *_= 0.001 and hence according to equation (15), *U*_*S *_= 0.499. Both (*U*_*S*_)^1-*α *^and (1RS)1−ρ
 MathType@MTEF@5@5@+=feaafiart1ev1aaatCvAUfKttLearuWrP9MDH5MBPbIqV92AaeXatLxBI9gBaebbnrfifHhDYfgasaacH8akY=wiFfYdH8Gipec8Eeeu0xXdbba9frFj0=OqFfea0dXdd9vqai=hGuQ8kuc9pgc9s8qqaq=dirpe0xb9q8qiLsFr0=vr0=vr0dc8meaabaqaciaacaGaaeqabaqabeGadaaakeaadaqadaqaamaalaaabaGaeGymaedabaGaemOuai1aaSbaaSqaaiabdofatbqabaaaaaGccaGLOaGaayzkaaWaaWbaaSqabeaacqaIXaqmcqGHsisliiGacqWFbpGCaaaaaa@3598@ now contribute to increase *W*_*A,S *_and *W*_*R,S *_respectively such that *W*_*A,S *_and *W*_*R,S *_have higher values in the current situation of near-minimum redundancy than in the previous situation of near-maximum redundancy. The value of (1RS)1−ρ
 MathType@MTEF@5@5@+=feaafiart1ev1aaatCvAUfKttLearuWrP9MDH5MBPbIqV92AaeXatLxBI9gBaebbnrfifHhDYfgasaacH8akY=wiFfYdH8Gipec8Eeeu0xXdbba9frFj0=OqFfea0dXdd9vqai=hGuQ8kuc9pgc9s8qqaq=dirpe0xb9q8qiLsFr0=vr0=vr0dc8meaabaqaciaacaGaaeqabaqabeGadaaakeaadaqadaqaamaalaaabaGaeGymaedabaGaemOuai1aaSbaaSqaaiabdofatbqabaaaaaGccaGLOaGaayzkaaWaaWbaaSqabeaacqaIXaqmcqGHsisliiGacqWFbpGCaaaaaa@3598@ for a large part of the domain of *ρ *(0 to 0.6) is at least in the order of 10, which is two orders of magnitude greater than the typical values of (*U*_*S*_)^1-*α *^and (*V*_*S*_)^*α *^in the same situation (between 0 and 1). Due to this chasm in magnitude, the contribution of (*V*_*S*_)^*α *^(regardless of its value) will be subjugated by that of (1RS)1−ρ
 MathType@MTEF@5@5@+=feaafiart1ev1aaatCvAUfKttLearuWrP9MDH5MBPbIqV92AaeXatLxBI9gBaebbnrfifHhDYfgasaacH8akY=wiFfYdH8Gipec8Eeeu0xXdbba9frFj0=OqFfea0dXdd9vqai=hGuQ8kuc9pgc9s8qqaq=dirpe0xb9q8qiLsFr0=vr0=vr0dc8meaabaqaciaacaGaaeqabaqabeGadaaakeaadaqadaqaamaalaaabaGaeGymaedabaGaemOuai1aaSbaaSqaaiabdofatbqabaaaaaGccaGLOaGaayzkaaWaaWbaaSqabeaacqaIXaqmcqGHsisliiGacqWFbpGCaaaaaa@3598@ for a large range of *ρ*. Conversely, due to the stable limits of (*U*_*S*_)^1-*α *^∈ (0,1], the contributions of (*U*_*S*_)^1-*α *^and (*V*_*S*_)^*α *^are almost counter-balanced by each other, with the exception of the extreme cases of *α *= 0 and *α *= 1 (Figure 7(c)).

### The *F*-splits evaluation procedure

In several previous studies on feature selection for microarray datasets [3-5], feature selection techniques have been applied *once *on the *full *dataset (with no splitting) before cross validation procedure (be it LOOCV or *n*-fold cross validation) is employed to evaluate the classification performance of the resulting predictor sets. We call this evaluation procedure the **i**nternal **c**ross **v**alidation (ICV) procedure. ICV is known to produce selection bias, which leads to overly optimistic estimates of accuracy [58]. In [59], datasets are explicitly split into training and test sets, but information from the test set is incorporated into the feature selection process – which results in over-fitting and again, overly optimistic estimates of accuracy.

To avoid this pitfall, different splits of the dataset into training and test sets should be used where feature selection is repeated for each of the splits. During each split, our feature selection techniques will be applied only on the training set of that particular split. No information from the test set should be 'leaked' into the process of forming the predictor set (which is precisely what happens during the ICV procedure). Classifier trained on the predictor set and the training samples will then be used to predict the class of the test samples of the current split. The test set accuracies obtained from each split will be averaged to give an estimate of the classification accuracy. We call this procedure of accuracy estimation the *F*-splits evaluation procedure (*F *being the number of splits used). Figure 1 shows how we evaluate the performance of each predictor set scoring method run at a certain value of the DDP at each value of *P*.

The selection bias resulting from the ICV procedure makes it possible for any feature selection technique evaluated using ICV to produce accuracies which are much higher than those from the *F*-splits evaluation procedure. The overly optimistic estimates of accuracy from the ICV mean that predictor sets chosen based on the ICV are likely to give dismal classification performance in the face of fresh, unknown test samples. The effect of selection bias on multiclass microarray datasets has been elucidated in [11].

### Summary of previous works

The concept of the DDP was first introduced in [57], where we proposed the antiredundancy-based *W*_*A,S *_scoring method. However, in that study, only one dataset (the GCM dataset [2]) was analyzed, and no *F*-splits evaluation procedure was implemented since only the original (single) split of training and test sets used in [2] was investigated. In a following study [29], we added the redundancy-based *W*_*R,S *_scoring method and made some simple comparisons between the two scoring methods (using overall accuracy and range of class accuracies). Again, only the single split of the GCM dataset was analyzed.

In our third study on the DDP feature selection techniques [11], five datasets were used as benchmark datasets: GCM [2], NCI60 [15], lung [21], MLL [23] and AML/ALL [25]. All five are included in the set of nine microarray datasets analyzed in this study. However, the study in [11] was limited to the *W*_*A,S *_scoring method. It was in the same study [11] that we first implemented the *F*-splits evaluation procedure and demonstrated the importance of avoiding evaluation procedures prone to producing overly optimistic estimates of accuracy. All of the details concerning the *W*_*A,S *_and the *W*_*R,S *_scoring methods, and the *F*-splits evaluation procedure are described in the **Methods **section of this paper.

In our most recent study [34], we presented a procedure for computing the value of the DDP most likely to produce the optimal accuracy based on available classification results. This procedure is elaborated in **The importance of the DDP **sub-section under the **Discussion **section of this paper.

## Authors' contributions

CHO designed and implemented the DDP-based feature selection techniques, and drafted the manuscript. MC supervised and SWT co-supervised the study. Both MC and SWT provided inputs on the algorithm implementation and substantial edits on the manuscript. All authors read and approved the final manuscript.

## Supplementary Material

Additional File 1 This compressed file contains codes (MATLAB M-files) for the DDP-based predictor set scoring methods and an example dataset. It is viewable using any archive utility such as WinZip or as a compressed folder in Windows XP. The MATLAB M-files are viewable using MATLAB Editor/Debugger. Click here for file

## Figures and Tables

**Table 1:** Descriptions of benchmark datasets.

Dataset	Type	*N*	*K*	Training:Test set size	*P*_max_
BRN	cDNA	7452	15	176:84	150
BRN14	cDNA	7452	14	174:83	150
GCM	Affymetrix	10820	14	144:54	150
NCI60	cDNA	7386	8	40:20	150
PDL	Affymetrix	12011	6	166:82	120
Lung	Affymetrix	1741	5	135:68	100
SRBC	cDNA	2308	4	55:28	80
MLL	Affymetrix	8681	3	48:24	60
AML/ALL	Affymetrix	3571	3	48:24	60

*N *is the number of features after preprocessing. *K *is the number of classes in the dataset.

**Table 2:** Best estimate of accuracy.

Dataset	*W*_*A,S*_	*W*_*R,S*_	Superior method
BRN	94.3%, *α *= 0.6	94.0%, *ρ *= 0.9	*W*_*A,S*_
BRN14	94.7%, *α *= 0.33	94.5%, *ρ *= 0.6	*W*_*A,S*_
GCM	80.2%, *α *= 0.4	80.2%, *ρ *= 0.5	Equal
NCI60	68.0%, *α *= 0.1	68.0%, *ρ *=0.6	Equal
PDL	98.4%, *α *=0.5	98.3%, *ρ *=1	*W*_*A,S*_
Lung	94.1%, *α *=0.4	93.8%, *ρ *=0.8	*W*_*A,S*_
SRBC	98.9%, *α *=0.73	98.9%, *ρ *=0.85	Equal
MLL	98.3%, *α *=0.3	97.9%, *ρ *=0.45	*W*_*A,S*_
AML/ALL	97.5%, *α *=0.5	97.1%, *ρ *=0.7	*W*_*A,S*_

Best estimate of accuracy from the *W*_*A,S *_and the *W*_*R,S *_scoring methods, obtained at *P *= *P*_max_, followed by the corresponding value of the DDP.

**Table 3:** Range of class accuracies.

Dataset	*W*_*A,S*_	*W*_*R,S*_	Superior method
BRN	0.80	0.80	Equal
BRN14	0.80	0.71	*W*_*R,S*_
GCM	0.81	0.83	*W*_*A,S*_
NCI60	0.68	0.85	*W*_*A,S*_
PDL	0.18	0.19	*W*_*A,S*_
Lung	0.35	0.32	*W*_*R,S*_
SRBC	0.03	0.03	Equal
MLL	0.05	0.05	*W*_*A,S*_
AML/ALL	0.05	0.06	*W*_*A,S*_

Range of class accuracies from the *W*_*A,S *_and the *W*_*R,S *_scoring methods, obtained using the values of DDP shown in Table 2 at *P *= *P.*

**Table 4:** Overall comparison of the *W*_*A,S *_and the *W*_*R,S *_scoring methods.

Dataset	Superior method based on best estimate of accuracy	Superior method based on range of class accuracies	Overall superior method
BRN	*W*_*A,S*_	Equal	*W*_*A,S*_
BRN14	*W*_*A,S*_	*W*_*R,S*_	Undecided
GCM	Equal	*W*_*A,S*_	*W*_*A,S*_
NCI60	Equal	*W*_*A,S*_	*W*_*A,S*_
PDL	*W*_*A,S*_	*W*_*A,S*_	*W*_*A,S*_
Lung	*W*_*A,S*_	*W*_*R,S*_	Undecided
SRBC	Equal	Equal	Equal
MLL	*W*_*A,S*_	*W*_*A,S*_	*W*_*A,S*_
AML/ALL	*W*_*A,S*_	*W*_*A,S*_	*W*_*A,S*_

Comparing the *W*_*A,S *_and the *W*_*R,S *_scoring methods through both best estimate of accuracy and range of class accuracies.

**Table 5:** Comparing the *W*_*A,S *_and the *W*_*R,S *_scoring methods through statistical tests.

Dataset	*A*	*C*
BRN	16	145
BRN14	33	145
GCM	18	145
NCI60	5	112
PDL	24	115
Lung	22	95
SRBC	10	75
MLL	7	55
AML/ALL	11	55

*A *is the number of times the null hypothesis that the *W*_*R,S *_scoring method is as good as the *W*_*A,S *_scoring method is rejected in favour of the *W*_*A,S *_scoring method (binomial test). *C *is the number of times that the right-sided *p*-value associated with the Wilcoxon signed rank test statistic is below the significance level of 0.05. The maximum values of *A *and *C *are both *P*_max_-1.

**Table 6:** Most frequently selected genes for the BRN dataset.

Rank	Annotation	Remarks	Group
1	TYR tyrosinase (oculocutaneous albinism IA)	Identified as marker for skin tumor class in [12]	M
2	FLJ20624 **hypothetical protein FLJ20624	Related to the gene PAK1, which is associated with pancreatic cancer [35]	1
3	DMXL1 Dmx-like 1	Function still unknown, although high level of conservation suggests important roles [36]	4
4	CLDN4 claudin 4	Identified as marker for ovarian, bladder, lung, and stomach tumor classes in [12]	M
5	TACSTD1 tumor-associated calcium signal transducer 1	Identified as marker for stomach, pancreatic, lung, and breast cancer classes in [12]	M
6	M6PR mannose-6-phosphate receptor (cation dependent)	Defective function of M6PR leads to hepatocellular carcinoma [37]	1
7	PLG plasminogen	Identified as marker for stomach cancer class in [12]	M
8	SPINT2 serine protease inhibitor, Kunitz type, 2	Found to be under-expressed in epithelial ovarian cancer patients [38]	1
9	SORD sorbitol dehydrogenase	Suppresses growth arrest induced by a p53 tumor mutant in fission yeast [39]	2
10	FGA fibrinogen, A alpha polypeptide	Mutation of FGA found in breast cancer patients [40]	1
11	BCL6 B-cell CLL/lymphoma 6 (zinc finger protein 51)	Deregulation of BCL6 found in diffuse large cell lymphoma [41]	1
12	APOH apolipoprotein H (beta-2-glycoprotein I)	Identified as marker for liver cancer class in [12]	M
13	PAX8 paired box gene 8	Verified as marker for ovarian cancer in [12], also identified as marker for renal and breast cancer classes in [12]	M
14	APCS amyloid P component, serum	Produces normal circulating plasma protein that is deposited on amyloid fibrils	4
15	S100A1 S100 calcium-binding protein A1	Identified as marker for breast, kidney, and ovary cancer classes in [12]	M
16	AMD1 S-adenosylmethionine decarboxylase 1	Specifically up-regulated in B cell lymphoma [42]	1
17	FABP1 fatty acid binding protein 1, liver	Identified as marker for pancreatic cancer class in [12]	M
18	HLCS holocarboxylase synthetase (biotin- [proprionyl-Coenzyme A-carboxylase (ATP-hydrolysing)] ligase)	The enzyme holocarboxylase synthetase plays a role in gene regulation (determining whether genes are turned on or off)	4
19	ITIH3 pre-alpha (globulin) inhibitor, H3 polypeptide	Its product is predominantly transcribed in liver, and is involved in pathological conditions such as tumor invasion and metastasis [43]	2
20	KRT18 keratin 18	Identified as marker for CNS (central nervous system) and stomach cancer classes in [12]	M
21	LGALS4 lectin, galactoside-binding, soluble, 4 (galectin 4)	Verified as marker for pancreatic cancer in [12], also identified as marker for stomach, liver, kidney, and breast cancer classes in [12]	M
22	HELO1 homolog of yeast long chain polyunsaturated fatty acid elongation enzyme 2	Highly expressed in adrenal gland and testis (tissue-specific), probably involved in encoding the major histocompatibility complex, essential to human immune response [44]	3
23	EST	Unknown sequence	4
24	QKI homolog of mouse quaking QKI (KH domain RNA binding protein)	Specifically expressed in the central nervous system (CNS)	3
25	NDP Norrie disease (pseudoglioma)	Regulates neural cell proliferation and differentiation. Norrie disease (caused by mutation of NDP) is also accompanied by intraocular tumor [45]	3

Top 25 genes ranked from the most frequently selected genes for the BRN dataset. Group M: identified as a marker or repressor of a specific tumor type in originating study. Group 1: identified as a marker or repressor of a specific tumor type in other studies. Group 2: known to either promote or inhibit tumor in general. Group 3: tissue-specific genes. Group 4: unknown sequences and genes with either still-unidentified function or general housekeeping roles.

**Table 7:** Most frequently selected genes for the BRN14 dataset.

Rank	Annotation	Remarks	Group
1	FLJ20624 **hypothetical protein FLJ20624	Gene #2 in the BRN dataset (Table 6)	1
2	M6PR mannose-6-phosphate receptor (cation dependent)	Gene #6 in the BRN dataset (Table 6)	1
3	PAX8 paired box gene 8	Gene #13 in the BRN dataset (Table 6)	M
4	DMXL1 Dmx-like 1	Gene #3 in the BRN dataset (Table 6)	4
5	PLG plasminogen	Gene #7 in the BRN dataset (Table 6)	M
6	LGALS4 lectin, galactoside-binding, soluble, 4 (galectin 4)	Gene #21 in the BRN dataset (Table 6)	M
7	APCS amyloid P component, serum	Gene #14 in the BRN dataset (Table 6)	4
8	GATA3 GATA-binding protein 3	Verified as marker for breast cancer in [12], also identified as marker for bladder cancer class in [12]	M
9	TACSTD1 tumor-associated calcium signal transducer 1	Gene #5 in the BRN dataset (Table 6)	M
10	FGA fibrinogen, A alpha polypeptide	Gene #10 in the BRN dataset (Table 6)	1
11	FABP1 fatty acid binding protein 1, liver	Gene #17 in the BRN dataset (Table 6)	M
12	SORD sorbitol dehydrogenase	Gene #9 in the BRN dataset (Table 6)	2
13	EST	Unknown sequence	4
14	DDOST dolichyl-diphosphooligosaccharide-protein glycosyltransferase	Identified as marker for testis cancer class in [12]	M
15	ITIH3 pre-alpha (globulin) inhibitor, H3 polypeptide	Gene #19 in the BRN dataset (Table 6)	2
16	KIAA0128 KIAA0128 protein; septin 2	Up-regulated in renal cell carcinoma [46]	1
17	BCL6 B-cell CLL/lymphoma 6 (zinc finger protein 51)	Gene #11 in the BRN dataset (Table 6)	1
18	AMD1 S-adenosylmethionine decarboxylase 1	Gene #16 in the BRN dataset (Table 6)	1
19	SERPINC1 serine (or cysteine) proteinase inhibitor, clade C (antithrombin), member 1	Controls expression of oncogene for hepatocarcinoma [47]	1
20	APOH apolipoprotein H (beta-2-glycoprotein I)	Gene #12 in the BRN dataset (Table 6)	M
21	HLCS holocarboxylase synthetase (biotin- [proprionyl-Coenzyme A-carboxylase (ATP-hydrolysing)] ligase)	Gene #18 in the BRN dataset (Table 6)	4
22	QKI homolog of mouse quaking QKI (KH domain RNA binding protein)	Gene #24 in the BRN dataset (Table 6)	3
23	Homo sapiens mRNA for putative nuclear protein (ORF1-FL49)	Expressed in spinal cord (high tissue-specificity)	3
24	GRHPR glyoxylate reductase/hydroxypyruvate reductase	One of the partners of the BCL6 (see Gene #11 in Table 6) translocation in follicular lymphoma, which leads to higher risk of transformation into aggressive lymphoma [48]	1
25	HELO1 homolog of yeast long chain polyunsaturated fatty acid elongation enzyme 2	Gene #22 in the BRN dataset (Table 6)	3

Top 25 genes ranked from the most frequently selected genes for the BRN14 dataset. Group M: identified as a marker or repressor of a specific tumor type in originating study. Group 1: identified as a marker or repressor of a specific tumor type in other studies. Group 2: known to either promote or inhibit tumor in general. Group 3: tissue-specific genes. Group 4: unknown sequences and genes with either still-unidentified function or general housekeeping roles.

**Table 8:** Most frequently selected genes for the GCM dataset.

Rank	Annotation	Remarks	Group
1	Human DNA sequence from clone 753P9 on chromosome Xq25-26.1. Contains the gene coding for Aminopeptidase P (EC 3.4.11.9, XAA-Pro/X-Pro/Proline/Aminoacylproline Aminopeptidase) and a novel gene.	Ranked #31 in the OVA marker list for the lymphoma class in [2]	M
2	Antigen, Prostate Specific, Alt. Splice Form 2	Ranked #8 in the OVA marker list for the prostate cancer class in [2]	M
3	Galectin-4	Ranked #1 in the OVA marker list for the colorectal cancer class in [2]	M
4	Homo sapiens mRNA for APCL protein, complete cds	Under-expressed in ovarian cancer, and thus a potential tumor suppressor gene in ovarian cancer [50]	1
5	Ins(1,3,4,5)P4-binding protein	Ranked #24 in the OVA marker list for the leukemia class in [2]	M
6	CARCINOEMBRYONIC ANTIGEN PRECURSOR	Ranked #4 in the OVA marker list for the colorectal cancer class in [2]	M
7	PMEL 17 PROTEIN PRECURSOR	Ranked #6 in the OVA marker list for the melanoma class in [2]	M
8	KLK1 Kallikrein 1 (renal/pancreas/salivary)	Ranked #18 in the OVA marker list for the prostate cancer class in [2]	M
9	EST: zt56g08.s1 Soares ovary tumor NbHOT Homo sapiens cDNA clone 726398 3', mRNA sequence. (from Genbank)	Ranked #5 in the OVA marker list for the mesothelioma class in [2]	M
10	EST: zr71g09.s1 Soares NhHMPu S1 Homo sapiens cDNA clone 668896 3', mRNA sequence. (from Genbank)	Ranked #5 in the OVA marker list for the CNS tumor class in [2]	M
11	Ribosomal protein S19	Ranked #1 in the OVA marker list for the lymphoma class in [2]	M
12	PULMONARY SURFACTANT-ASSOCIATED PROTEIN B PRECURSOR	Ranked #3 in the OVA marker list for the lung cancer class in [2]	M
13	Mammaglobin 2	A marker for breast cancer [51]	1
14	MRJ gene for a member of the DNAJ protein family	Associated with a tumor-transforming gene protein [52]	2
15	LPAP gene	Its product mediates proliferative and/or morphologic effects on ovarian cancer cells [53]	1
16	EST: zq49c07.s1 Stratagene hNT neuron (#937233) Homo sapiens cDNA clone 633036 3', mRNA sequence. (from Genbank)	Identified as a marker of CNS tumor in [54]	1
17	Eyes absent homolog (Eab1) mRNA	Up-regulated in epithelial ovarian cancer [55]	1
18	Pulmonary surfactant-associated protein SP-A (SFTP1) gene	Ranked #1 in the OVA marker list for the lung cancer class in [2]	M
19	EST: ab17g09.s1 Stratagene lung (#937210) Homo sapiens cDNA clone 841120 3' similar to contains LTR7.b2 LTR7 repetitive element ;, mRNA sequence. (from Genbank)	Ranked #10 in the OVA marker list for the lung cancer class in [2]	M
20	TUMOR-ASSOCIATED ANTIGEN CO-029	Ranked #5 in the OVA marker list for the colorectal cancer class in [2]	M
21	MLANA Differentiation antigen melan-A	Ranked #4 in the OVA marker list for the melanoma class in [2]	M
22	LI-cadherin	Ranked #3 in the OVA marker list for the colorectal cancer class in [2]	M
23	Antigen, Prostate Specific, Alt. Splice Form 3	Ranked #39 in the OVA marker list for the prostate cancer class in [2]	M
24	GPX2 Glutathione peroxidase 2, gastrointestinal	Found to play a role in colon cancer resistance [56]	1
25	Phosphodiesterase 9A	Ranked #28 in the OVA marker list for the prostate cancer class in [2]	M

Top 25 genes ranked from the most frequently selected genes for the GCM dataset. Group M: identified as a marker or repressor of a specific tumor type in originating study. Group 1: identified as a marker or repressor of a specific tumor type in other studies. Group 2: known to either promote or inhibit tumor in general. Group 3: tissue-specific genes. Group 4: unknown sequences and genes with either still-unidentified function or general housekeeping roles.

## References

[B1] Li T, Zhang C, Ogihara M (2004). A comparative study of feature selection and multiclass classification methods for tissue classification based on gene expression. Bioinformatics.

[B2] Ramaswamy S, Tamayo P, Rifkin R, Mukherjee S, Yeang CH, Angelo M, Ladd C, Reich M, Latulippe E, Mesirov JP, Poggio T, Gerald W, Loda M, Lander ES, Golub TR (2001). Multi-class cancer diagnosis using tumor gene expression signatures. Proc Natl Acad Sci USA.

[B3] Chai H, Domeniconi C (2004). An evaluation of gene selection methods for multi-class microarray data classification. Proceedings of the Second European Workshop on Data Mining and Text Mining in Bioinformatics.

[B4] Ding C, Peng H (2003). Minimum redundancy feature selection from microarray gene expression data. Proceedings of the Second IEEE Computational Systems Bioinformatics Conference.

[B5] Yu L, Liu H (2004). Redundancy based feature selection for microarray data. Proceddings of the 2004 ACM SIGKDD.

[B6] Liu JJ, Cutler G, Li W, Pan Z, Peng S, Hoey T, Chen L, Ling XB (2005). Multiclass cancer classification and biomarker discovery using GA-based algorithms. Bioinformatics.

[B7] Jirapech-Umpai T, Aitken S (2005). Feature selection and classification for microarray data analysis: Evolutionary methods for identifying predictive genes. BMC Bioinformatics.

[B8] Hall MA, Smith LA (1998). Practical feature subset selection for machine learning. Proceedings of the 21st Australasian Computer Science Conference.

[B9] Guyon I, Elisseeff A (2003). An introduction to variable and feature selection. J Machine Learning Res.

[B10] Knijnenburg TA (2004). Selecting relevant and non-redundant features in microarray classification applications. MSc Thesis.

[B11] Ooi CH, Chetty M, Teng SW, Oliveira JL, Maojo V, Martín-Sánchez F, Pereira, AS (2005). Relevance, redundancy and differential prioritization in feature selection for multiclass gene expression data. Proceedings of the Sixth International Symposium on Biological and Medical Data Analysis.

[B12] Munagala K, Tibshirani R, Brown P (2004). Cancer characterization and feature set extraction by discriminative margin clustering. BMC Bioinformatics.

[B13] Discriminative Margin Clustering. http://microarray-pubs.stanford.edu/margin_clus/.

[B14] Broad Institute Cancer Program Publications. http://www.broad.mit.edu/cgi-bin/cancer/publications/pub_paper.cgi?mode=view&paper_id=61.

[B15] Ross DT, Scherf U, Eisen MB, Perou CM, Rees C, Spellman P, Iyer V, Jeffrey SS, Van de Rijn M, Waltham M, Pergamenschikov A, Lee JCF, Lashkari D, Shalon D, Myers TG, Weinstein JN, Botstein D, Brown PO (2000). Systematic variation in gene expression patterns in human cancer cell lines. Nat Genet.

[B16] Stanford NCI60 Cancer Microarray Project. http://genome-www.stanford.edu/nci60/.

[B17] Yeoh E-J, Ross ME, Shurtleff SA, Williams WK, Patel D, Mahfouz R, Behm FG, Raimondi SC, Relling MV, Patel A, Cheng C, Campana D, Wilkins D, Zhou X, Li J, Liu H, Pui C-H, Evans WE, Naeve C, Wong L, Downing JR (2002). Classification, subtype discovery, and prediction of outcome in pediatric lymphoblastic leukemia by gene expression profiling. Cancer Cell.

[B18] St. Jude Research/Supplemental Data/ALL1/Data Files. http://www.stjuderesearch.org/data/ALL1/all_datafiles.html.

[B19] Khan J, Wei JS, Ringner M, Saal LH, Ladanyi M, Westermann F, Berthold F, Schwab M, Antonescu CR, Peterson C, Meltzer PS (2001). Classification and diagnostic prediction of cancers using expression profiling and artificial neural networks. Nat Med.

[B20] Microarray Project. http://research.nhgri.nih.gov/microarray/Supplement/.

[B21] Bhattacharjee A, Richards WG, Staunton JE, Li C, Monti S, Vasa P, Ladd C, Beheshti J, Bueno R, Gillette M, Loda M, Weber G, Mark EJ, Lander ES, Wong W, Johnson BE, Golub TR, Sugarbaker DJ, Meyerson M (2001). Classification of human lung carcinomas by mRNA expression profiling reveals distinct adenocarcinoma subclasses. Proc Natl Acad Sci USA.

[B22] Broad Institute Cancer Program Publications. http://www.broad.mit.edu/cgi-bin/cancer/publications/pub_paper.cgi?mode=view&paper_id=62.

[B23] Armstrong SA, Staunton JE, Silverman LB, Pieters R, den Boer ML, Minden MD, Sallan SE, Lander ES, Golub TR, Korsmeyer SJ (2002). MLL translocations specify a distinct gene expression profile that distinguishes a unique leukemia. Nat Genet.

[B24] Broad Institute Cancer Program Publications. http://www.broad.mit.edu/cgi-bin/cancer/publications/pub_paper.cgi?mode=view&paper_id=63.

[B25] Golub TR, Slonim DK, Tamayo P, Huard C, Gaasenbeek M, Mesirov JP, Coller H, Loh ML, Downing JR, Caligiuri MA, Bloomfield CD, Lander ES (1999). Class discovery and class prediction by gene expression monitoring. Science.

[B26] Broad Institute Cancer Program Publications. http://www.broad.mit.edu/cgi-bin/cancer/publications/pub_paper.cgi?mode=view&paper_id=43.

[B27] Dudoit S, Fridlyand J, Speed T (2002). Comparison of discrimination methods for the classification of tumors using gene expression data. J Am Stat Assoc.

[B28] Platt J, Cristianini N, Shawe-Taylor J (2000). Large margin DAGs for multiclass classification. Advances in Neural Information Processing Systems.

[B29] Ooi CH, Chetty M (2005). A Comparative Study of Two Novel Predictor Set Scoring Methods. Proceedings of the Sixth International Conference on Intelligent Data Engineering and Automated Learning (IDEAL-05).

[B30] Salzberg S (1997). On comparing classifiers: Pitfalls to avoid and a recommended approach. Data Mining and Knowledge Discovery.

[B31] Wilcoxon F (1945). Individual comparisons by ranking methods. Biometrics.

[B32] Linder R, Dew D, Sudhoff H, Theegarten D, Remberger K, Poppl SJ, Wagner M (2004). The subsequent artificial neural network (SANN) approach might bring more classificatory power to ANN-based DNA microarray analyses. Bioinformatics.

[B33] Park M, Hastie T (2005). Hierarchical classification using shrunken centroids. Technical Report.

[B34] Ooi CH, Chetty M, Teng SW, Simoff SJ, Williams GJ, Galloway J, Kolyshkina I (2005). Modeling Microarray Datasets for Efficient Feature Selection. Proceedings of the 4th Australasian Conference on Knowledge Discovery and Data Mining (AusDM05).

[B35] Hirokawa Y, Levitzki A, Lessene G, Baell J, Xiao C, Zhu H, Maruta H (2003). Signal therapy of human pancreatic cancer and NF1-deficient breast cancer xenograft in mice by a combination of PP1 and GL- anti-PAK1 drugs (Tyr-kinase inhibitors). Cancer Letters.

[B36] Kraemer C, Enklaar T, Zabel B, Schmidt ER (2000). Mapping and structure of DMXL1, a human homologue of the DmX gene from Drosophila melanogaster coding for a WD repeat protein. Genomics.

[B37] Scharf JG, Dombrowski F, Ramadori G (2001). The IGF axis and hepatocarcinogenesis. Mol Pathol.

[B38] Wang X, Wang E, Kavanagh JJ, Freedman RS (2005). Ovarian cancer, the coagulation pathway, and inflammation. J Transl Med.

[B39] Wagner P, Grimaldi M, Jenkins JR (1993). Putative dehydrogenase tms1 suppresses growth arrest induced by a p53 tumour mutant in fission yeast. Eur J Biochem.

[B40] Mayall F, Fairweather S, Wilkins R, Chang B, Nicholls R (1999). Microsatellite abnormalities in plasma of patients with breast carcinoma: Concordance with the primary tumour. J Clin Pathol.

[B41] Chang C-C, Ye BH, Chagantit RSK, Dalla-Favera R (1996). BCL-6, a POZ/zinc-finger protein, is a sequence-specific transcriptional repressor. Proc Natl Acad Sci USA.

[B42] Marinkovic D, Marinkovic T, Kokai E, Barth T, Moller P, Wirth T (2004). Identification of novel Myc target genes with a potential role in lymphomagenesis. Nucleic Acids Research.

[B43] Ruminy P, Rouet P, Salier J-P (2003). An interplay of Sp1, GKLF and CREB-2 controls human Pre-α-Inhibitor gene (ITIH3) transcription. Gene.

[B44] Leonard AE, Bobik EG, Dorado J, Kroeger PE, Chuang L-T, Thurmond JM, Parker-Barnes JM, Das T, Huang Y-S, Mukerji P (2000). Cloning of a human cDNA encoding a novel enzyme involved in the elongation of long-chain polyunsaturated fatty acids. Biochem J.

[B45] Fuchs S, Kellner U, Wedemann H, Gal A (1995). Missense mutation (Arg121Trp) in the Norrie disease gene associated with X-linked exudative vitreoretinopathy. Hum Mutat.

[B46] Craven RA, Stanley AJ, Hanrahan S, Dods J, Unwin R, Totty N, Harnden P, Eardley I, Selby PJ, Banks RE Proteomic analysis of primary cell lines identifies protein changes present in renal cell carcinoma. Proteomics.

[B47] Dubois N, Bennoun M, Allemand I, Molina T, Grimber G, Daudet-Monsac M, Abelanet R, Briand P (1991). Time-course development of differentiated hepatocarcinoma and lung metastasis in transgenic mice. J Hepatol.

[B48] Akasaka T, Lossos IS, Levy R (2003). BCL6 gene translocation in follicular lymphoma: A harbinger of eventual transformation to diffuse aggressive lymphoma. Blood.

[B49] OVA_MARKERS. http://www.broad.mit.edu/mpr/publications/projects/Global_Cancer_Map/OVA_MARKERS.xls.

[B50] Jarrett CR, Blancato J, Cao T, Bressette DS, Cepeda M, Young PE, King CR, Byers SW (2001). Human APC2 localization and allelic imbalance. Cancer Research.

[B51] Fleming TP, Watson MA (2000). Mammaglobin, a breast-specific gene, and its utility as a marker for breast cancer. Ann N Y Acad Sci.

[B52] Lin P (1999). Pituitary tumor-transforming gene protein associates with ribosomal protein S10 and a novel human homologue of DnaJ in testicular cells. J Biol Chem.

[B53] Sun W-S, Imai A, Sugiyama M, Furui T, Tamaya T, Saio M, Morris AJ (2004). Translocation of lysophosphatidic acid phosphatase in response to gonadotropin-releasing hormone to the plasma membrane in ovarian cancer cell. American Journal of Obstetrics and Gynecology.

[B54] Antonov AV, Tetko IV, Mader MT, Budczies J, Mewes HW (2004). Optimization models for cancer classification: Extracting gene interaction information from microarray expression data. Bioinformatics.

[B55] Zhang L, Yang N, Huang J, Buckanovich RJ, Liang S, Barchetti A, Vezzani C, O'Brien-Jenkins A, Wang J, Ward MR, Courreges MC, Fracchioli S, Medina A, Katsaros D, Weber BL, Coukos G (2005). Transcriptional coactivator Drosophila eyes absent homologue 2 is up-regulated in epithelial ovarian cancer and promotes tumor growth. Cancer Research.

[B56] Mork H, Lex B, Scheurlen M, Dreher I, Schutze N, Kohrle J, Jakob F (1998). Expression pattern of gastrointestinal selenoproteins – targets for selenium supplementation. Nutr Cancer.

[B57] Ooi CH, Chetty M, Gondal I (2004). The role of feature redundancy in tumor classification. Proceedings of the International Conference on Bioinformatics and its Applications (ICBA'04).

[B58] Ambroise C, McLachlan GJ (2002). Selection bias in gene extraction on the basis of microarray gene-expression data. Proc Natl Acad Sci USA.

[B59] Ooi CH, Tan P (2003). Genetic algorithms applied to multi-class prediction for the analysis of gene expression data. Bioinformatics.

